# Improving the estimation of rice above-ground biomass based on spatio-temporal UAV imagery and phenological stages

**DOI:** 10.3389/fpls.2024.1328834

**Published:** 2024-05-07

**Authors:** Yan Dai, Shuang’en Yu, Tao Ma, Jihui Ding, Kaiwen Chen, Guangquan Zeng, Airong Xie, Pingru He, Suhan Peng, Mengxi Zhang

**Affiliations:** ^1^ College of Agricultural Science and Engineering, Hohai University, Nanjing, China; ^2^ Jiangsu Province Engineering Research Center for Agricultural Soil-Water Efficient Utilization, Carbon Sequestration and Emission Reduction, Nanjing, China; ^3^ College of Innovation and Entrepreneurship, Hunan Polytechnic of Water Resources and Electric Power, Changsha, China

**Keywords:** multispectral remote sensing, rice above-ground biomass, vegetation indices, digital surface model, plant height, canopy cover, regression method

## Abstract

**Introduction:**

Unmanned aerial vehicles (UAVs) equipped with visible and multispectral cameras provide reliable and efficient methods for remote crop monitoring and above-ground biomass (AGB) estimation in rice fields. However, existing research predominantly focuses on AGB estimation based on canopy spectral features or by incorporating plant height (PH) as a parameter. Insufficient consideration has been given to the spatial structure and the phenological stages of rice in these studies. In this study, a novel method was introduced by fully considering the three-dimensional growth dynamics of rice, integrating both horizontal (canopy cover, CC) and vertical (PH) aspects of canopy development, and accounting for the growing days of rice.

**Methods:**

To investigate the synergistic effects of combining spectral, spatial and temporal parameters, both small-scale plot experiments and large-scale field testing were conducted in Jiangsu Province, China from 2021 to 2022. Twenty vegetation indices (VIs) were used as spectral features, PH and CC as spatial parameters, and days after transplanting (DAT) as a temporal parameter. AGB estimation models were built with five regression methods (MSR, ENet, PLSR, RF and SVR), using the derived data from six feature combinations (VIs, PH+CC, PH+CC+DAT, VIs+PH +CC, VIs+DAT, VIs+PH+CC+DAT).

**Results:**

The results showed a strong correlation between extracted and ground-measured PH (R2 = 0.89, RMSE=5.08 cm). Furthermore, VIs, PH and CC exhibit strong correlations with AGB during the mid-tillering to flowering stages. The optimal AGB estimation results during the mid-tillering to flowering stages on plot data were from the PLSR model with VIs and DAT as inputs (*R* ^2^ = 0.88, *RMSE*=1111kg/ha, *NRMSE*=9.76%), and with VIs, PH, CC, and DAT all as inputs (*R* ^2^ = 0.88, *RMSE*=1131 kg/ha, *NRMSE*=9.94%). For the field sampling data, the ENet model combined with different feature inputs had the best estimation results (%error=0.6%–13.5%), demonstrating excellent practical applicability.

**Discussion:**

Model evaluation and feature importance ranking demonstrated that augmenting VIs with temporal and spatial parameters significantly enhanced the AGB estimation accuracy. In summary, the fusion of spectral and spatio-temporal features enhanced the actual physical significance of the AGB estimation models and showed great potential for accurate rice AGB estimation during the main phenological stages.

## 1 Introduction

Rice, as one of the most important staple crops in China, plays a pivotal role in ensuring food security and promoting agricultural sustainability (Huang et al., 2011; Wang et al., 2014). The above-ground biomass (AGB) serves as a key indicator for reflecting rice growth and predicting the yield (Confalonieri et al., 2009b; Choi et al., 2013; Adeluyi et al., 2022). Therefore, obtaining precise AGB estimates during the primary phenological stages of rice is essential for assessing rice growth status, targeting rice management, and optimizing agricultural practices (Gnyp et al., 2013; Zhang et al., 2017).

Conventional methods for obtaining rice growth parameters involve labor-intensive field measurements using various tools (Yao et al., 2013). Although these methods provide relatively accurate data, they are time-consuming and impractical for large-scale measurements (Confalonieri et al., 2009a). Notably, destructive sampling is required for direct AGB measurement, making it unsuitable for long-term monitoring and affecting yield assessment (Borra-Serrano et al., 2019; Nakajima et al., 2023). Also, it is difficult to accurately assess the overall condition of large rice fields due to the limitation of sampling points and spatial heterogeneity (Zhou et al., 2023). In recent years, remote sensing monitoring technologies, based on platforms such as satellites, airborne, unmanned aerial vehicles (UAVs) and ground, have emerged as innovative alternatives for AGB estimation (Duan et al., 2019; Mostofialmamaleki et al., 2019). These platforms are equipped with various sensors, including digital, multispectral, hyperspectral, thermal infrared and synthetic aperture radar (SAR), enabling timely and non-destructive acquisition of canopy information (Chakraborty et al., 1997; Mashimbye et al., 2012; Han et al., 2021; Qiu et al., 2021).

Satellite remote sensing possesses the capability for all-weather, multisource, and multiscale monitoring, allowing synchronous observations over large rice areas (Inoue et al., 2014; Wang et al., 2016; Jiang et al., 2021). However, it is accompanied by drawbacks such as long revisit periods, high satellite costs, and significant interference from cloud cover, posing challenges in ensuring monitoring precision (Feng et al., 2021). The combination of airborne hyperspectral and LiDAR data enables effective monitoring of biomass, but the cost of operating and maintaining airborne platforms is also high, making them more commonly used for large-scale forest monitoring than for crops such as rice (Brovkina et al., 2017; Gao et al., 2022). Ground remote sensing systems are close to the surface, facilitating more detailed information on rice, but are limited in their coverage due to fixed station locations (Gnyp et al., 2012). In comparison, the application of near-ground UAVs provides significant assistance in improving the temporal and spatial resolution of rice monitoring, with the advantages of cost-effectiveness, operational simplicity, and high efficiency in large-scale monitoring (Wan et al., 2020; Bascon et al., 2022; Luo et al., 2022; Xu et al., 2022a). In particular, consumer-grade UAVs equipped with visible and multispectral cameras can conveniently obtain canopy structural parameters and key spectral, color and texture features, which have been widely used to estimate AGB (Xu et al., 2022b; Zheng et al., 2023).

Traditional methods for estimating AGB from UAV imagery have mainly relied on vegetation indices (VIs), which have achieved acceptable estimation accuracy (Gnyp et al., 2014; Cheng et al., 2017). Researchers have developed dozens of VIs, such as Normalized difference vegetation index (NDVI), Leaf Chlorophyll Index (LCI) and Soil Adjusted Vegetation Index (SAVI) (Huete, 1988; Peñuelas et al., 1997; Xue and Su, 2017). These dimensionless indices integrate spectral data from different narrow-band wavelengths and can reflect characteristics including coverage, chlorophyll content, moisture, and health status (Bannari et al., 1995). Along with VIs that make use of spectral features, texture indices that reflect subtle variations in canopy structure are also commonly used to enhance the estimation of AGB (Wang et al., 2022b; Liu et al., 2023a). Many studies predominantly focus on establishing empirical relationships directly between these features and ground-measured AGB values using statistical analysis and machine learning algorithms. Typical methods involve Linear Regression, Partial Least Squares Regression, Support Vector Machine, Random Forest, and Artificial Neural Network (Yang et al., 2018; Zhang et al., 2020; Wang et al., 2023a, Wang et al., 2023b). The lack of a solid foundation in physics and physiology will limit the improvement of AGB estimation accuracy. Moreover, crop spectral response can be influenced by various factors, including crop type, moisture content, nutrient status, soil, and atmospheric spatio-temporal variations (Hoffer, 1978; Bannari et al., 1995). Therefore, VIs tend to be unstable during the long growth process of rice and have the potential for saturation under high biomass conditions, posing difficulties for AGB estimation over multiple phenological periods (Yue et al., 2019; Liu et al., 2023c).

To address the limitations of VIs, researchers have integrated plant height (PH) to enhance AGB estimation (Bendig et al., 2015; Panday et al., 2020; Wang et al., 2022a; Yang et al., 2023). Three-dimensional point cloud data of the crop canopy can be obtained using photogrammetry or LiDAR technology (Sun et al., 2022). After interpolation, the 3D point cloud data can be generated into a digital surface model (DSM) for extracting plant height. This method has been widely applied to UAV remote sensing monitoring of crop PH with strong physical significance and high accuracy (Kawamura et al., 2020; Ji et al., 2022; Liu et al., 2022c). However, relying solely on PH cannot comprehensively reflect rice growth as it involves only one dimension. To gain a more comprehensive understanding of changes during crop growth, Canopy Coverage (CC) has received attention from researchers (Walter et al., 2015; Shu et al., 2023). Canopy cover refers to the proportion of land covered by the vertical projection of the vegetation canopy (Guevara-Escobar et al., 2005; Lee and Lee, 2011), which can be used to quantify the expansion of the rice canopy in the horizontal dimension as AGB increases. Studies have shown that CC is a reliable parameter for reflecting plant canopy growth and estimating AGB, Leaf Area Index (LAI), and yield (Nielsen et al., 2012; Goodwin et al., 2018; García-Martínez et al., 2020). Currently, there are relatively few studies combining PH and CC for AGB estimation, emphasizing the need to strengthen the role of CC. The physical significance of AGB estimation can be further improved from both vertical and horizontal perspectives.

The stages of crop growth can be quantified through various metrics, including time-based measures like Days After Sowing (DAS) and thermal-based measures like Growing Degree Day (GDD) (Li et al., 2022). Additionally, widely adopted classification systems like the Feekes code and Zadoks code delineate distinct phenological stages of crops (Zadoks et al., 1974; Simmons et al., 1985; Chin et al., 1991). Connections can be established between these orderly digitized metrics and the accumulation of crop biomass. Typically, rice biomass can be effectively expressed as a function of time (t) using logistic or Gompertz models (Yu et al., 2002; Varela et al., 2021). Currently, studies have mainly focused on establishing AGB estimation models in specific or overall phenological stages of rice. Among them, the quantitative value for rice growth has received limited attention. Considering the effect of temporal parameters would compensate for the possible saturation and instability of spectral features as the crop grows. Therefore, apart from spectral and spatial parameters, the days after transplanting (DAT) of rice was also introduced as a parameter in this study to investigate its impact on AGB estimation across the main phenological stages. This integrated approach aims to provide more accurate and detailed information for monitoring AGB in rice growth, offering robust support for agricultural management and decision-making.

Considering the limitations of the commonly used methods for estimating rice AGB using VIs and PH, a novel multidimensional approach was introduced in this study that considers both spatial structure and phenological stages. In addition, to ensure the robustness and practical applicability of AGB estimation model, it was imperative to transition from the controlled small-scale experimental plots to the complex reality of large-scale rice fields. The combination of experimental and sampling data could reinforce the reliability of the models, making it a valuable tool for rice monitoring. The main objectives of this study were to (1) obtain 3D point cloud data and multispectral features of rice canopies by UAV-borne digital and multispectral cameras, (2) precisely extract 20 types of VIs, PH and CC based on UAV images from rice fields, (3) estimate rice AGB across main phenological stages with five regression algorithms (MSR, ENet, PLSR, RF and SVR) and six feature combinations (VIs, PH+CC, PH+CC+DAT, VIs+PH+CC, VIs+DAT, VIs+PH+CC+DAT) and evaluate the simulation and accuracy, (4) construct a multidimensional AGB estimation model that integrates spectral, temporal and spatial features of rice.

## 2 Materials and methods

### 2.1 Study area and experimental design

**Figure 1:**
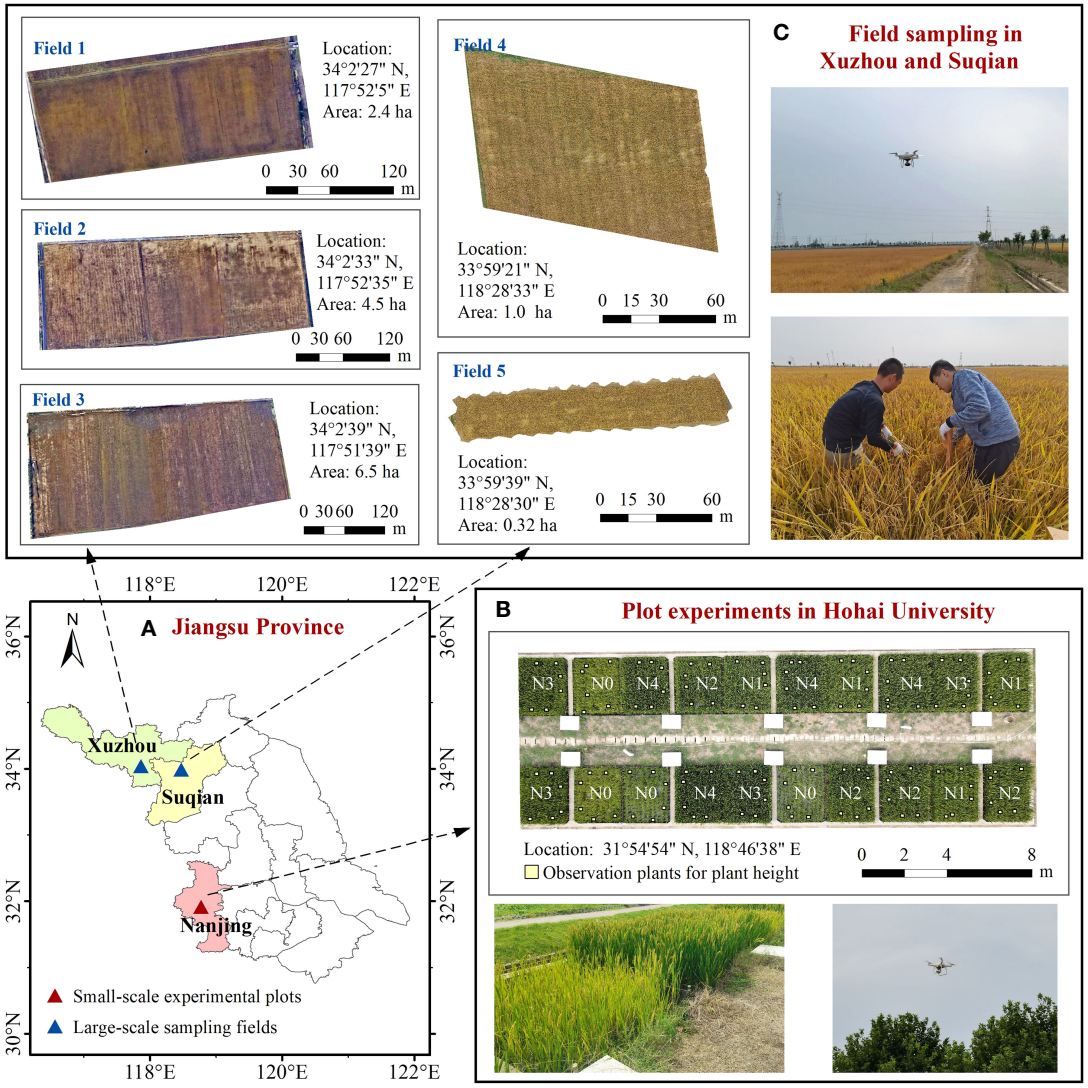
Layout of the small-scale experimental plots and large-scale sampling fields in this study. **(A)** Location of the plots and fields in Jiangsu Province. **(B)** Plot experiments in Hohai University. **(C)** Field sampling in Xuzhou (Field 1, Field 2 and Field 3) and Suqian (Field 4 and Field 5).

In this study, small-scale plot experiments and large-scale field sampling were both carried out to validate and assess the applicability of the AGB estimation models developed. The specific scheme is illustrated in **Figure 1**. The plot experiments were conducted at the experimental site of Hohai University in Nanjing, Jiangsu Province, China (31°55′N, 118°47′E) during 2021 and 2022. The study area was under a humid subtropical climate with an average annual precipitation of 1090.4mm and an average annual temperature of 15.4°C. Japonica rice variety Nanjing 9108 was transplanted into drainage lysimeters at a spacing of 20 cm × 15 cm. All plots were 2.5 meters long, 2 meters wide and 2 meters deep. Five nitrogen fertilizer treatments were used: 0 kg/ha (N0), 150 kg/ha (N1), 225 kg/ha (N2), 300 kg/ha (N3), and 375 kg/ha (N4), each with four replications. In addition, all treatments were applied with 75 kg/ha of phosphate fertilizer and 120 kg/ha of potash fertilizer. Nitrogen fertilizer was applied in three stages (base, tiller, and spike) at a 4:3:3 ratio, while phosphate fertilizer was applied as a base, and potash fertilizer was split into a base and spike application at a 1:1 ratio. Weeds in the plots were manually removed as appropriate.

To evaluate the AGB estimation models, extensive field sampling occurred in the northern region of Jiangsu Province in October 2021. This field sampling involved multiple areas, including three fields in Xuzhou (34°2′N, 117°52′E) and two fields in Suqian (33°59′N, 118°28′E), as depicted in **Figure 1C**. Both Xuzhou and Suqian experience a semi-humid monsoon climate, with an annual precipitation of about 900 mm and an average annual temperature of about 15°C. The same variety of japonica rice was also transplanted locally at a density of 300,000 plants per hectare.

### 2.2 Ground data acquisition

Rice plant height (PH) was measured as the vertical height from the soil surface to the top of the canopy using a steel ruler. In plot experiments, six rice plants were selected in each plot for periodic observation to assess the extracted plant height values from the UAV images. The acquisition time of ground data in plot experiments is shown in **Table 1**. On the day of each destructive sampling, we collected above-ground portions from three representative rice plants in the lysimeter plots and five representative rice plants from the fields. Subsequently, the PH of these plants was measured using the same steel ruler. The above-ground biomass (AGB) of these plants was then determined using the oven-drying method. These plant samples were initially dried at a constant temperature of 105°C for 0.5 hours, followed by a constant temperature of 75°C for 48 hours until reaching a constant temperature. The dry weight of each sample was measured using an electronic balance. For plot experiments, the average AGB of three plant samples per plot, after multiplied by the rice planting density, was converted into the average AGB per hectare (kg/ha). From a total of 10 samplings over two years, 200 groups of data were obtained in plot experiments. For field sampling, the average AGB of five plant samples from each of the five fields was calculated as individual plants (g/plant).

**Table 1:** Ground data measurement in plot experiments.

Year	Transplanting date	Harvest date	Indicator	Date of measurement
2021	3 July	25 October	AGB, PH	Jointing-booting stage: DAT48
				Milk ripening stage: DAT69
				Yellow ripening stage: DAT114
2022	26 June	22 October	AGB, PH	Tillering stage: DAT15, DAT27, DAT42
				Jointing-booting stage: DAT52
				Heading-flowering stage: DAT68
				Milk ripening stage: DAT82
				Yellow ripening stage: DAT109
			PH	Tillering stage: DAT30, DAT40
				Jointing-booting stage: DAT50
				Heading-flowering stage: DAT60
				Milk ripening stage: DAT70

AGB, above-ground biomass; PH, plant height; DAT, days after transplanting.

### 2.3 UAV data acquisition and processing

#### 2.3.1 Image acquisition and compositing

The DJI Phantom 4-Multispectral (P4M) (DJ-Innovations, Shenzhen, China) was employed for this experiment, featuring an integrated multispectral imaging system comprising one visible light camera and five multispectral cameras (Blue, Green, Red, Red Edge, and NIR). These cameras were responsible for capturing visible light and multispectral images, respectively, with the single camera boasting an effective pixel count of 2.08 million. Additionally, the DJI P4M was equipped with a Real-Time Kinematic (RTK) system, ensuring centimeter-level positioning accuracy. In positioning mode, the DJI P4M could fly at a maximum horizontal speed of 50 km/h. The vertical hovering accuracy was ±0.1 m and the horizontal hovering accuracy was ±0.1 m.

All image acquisitions were conducted during midday hours (10:00 to 14:00) on clear, cloudless or less cloudy days. Flight missions were planned using the software DJI GS Pro (DJ-Innovations, Shenzhen, China), with a forward overlap rate of 80% and a side overlap of 70%. The DJI P4M captured images in “waypoint hovering” mode and flew at 18 km/h. The altitude for each mission of the plot experiments and field sampling was set at 10m and 35m, respectively. The corresponding image accuracies were 0.5 cm/px and 1.9 cm/px, respectively. Two calibration boards of known reflectance were also photographed each time the UAV mission was conducted for later radiometric correction of the images. The reflectance of calibration boards in different bands is shown in **Table 2**.

**Table 2:** Multispectral spectrum and reflectance of calibration boards.

Band name	Wavelength	Reflectance of calibration boards	
		25%	50%
Blue	450 ± 16nm	0.2602	0.4771
Green	560 ± 16nm	0.2613	0.488
Red	650 ± 16nm	0.251	0.4949
Red Edge	730 ± 16nm	0.2465	0.4957
NIR	840 ± 26nm	0.2482	0.5005

**Figure 2:**
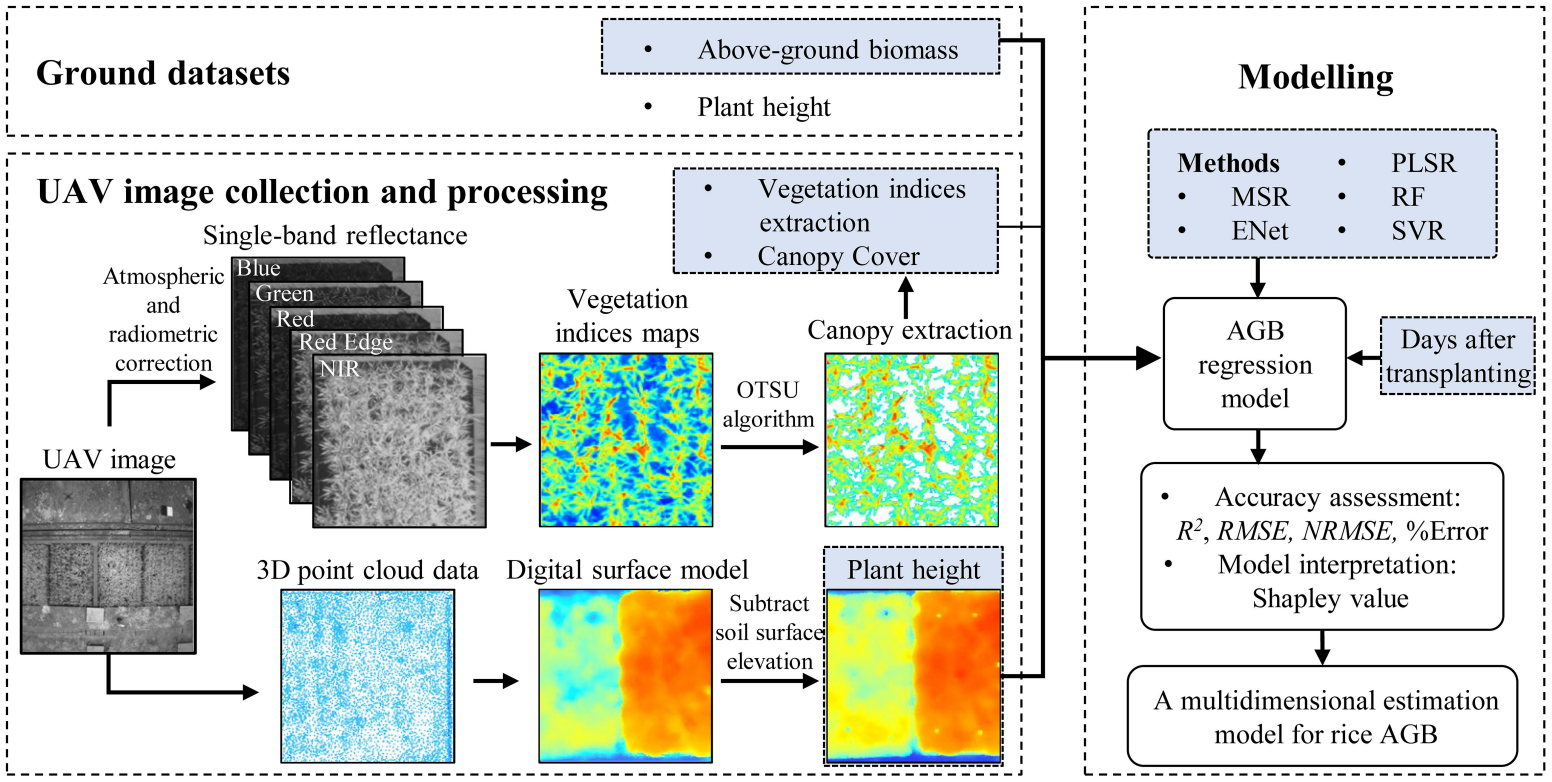
The main methods of this study.

Before transplanting the rice, and on the day of PH measurement and destructive sampling, UAV missions were conducted to obtain images. All UAV images were reconstructed in 2D multispectral by the software DJI Terra (DJ-Innovations, Shenzhen, China). The 3D point cloud data was rasterized to obtain the DSM. The calibration data was imported for reflectance radiometric correction to generate ortho-mosaic results for each band (**Figure 2**).

#### 2.3.2 Calculation of vegetation indices, plant height and canopy cover

The elevation data of the images at different growth stages were uniformly corrected by setting fixed control points. Rice PH at different stages was extracted by subtracting the DSM of the rice canopy from the bare soil DSM using the Raster Calculator function of ArcMap 10.8. At the corresponding position of each designated observation plant, a 10 cm × 10 cm sample square was drawn as a polygon element. Then the mean value of all raster data within the frame was calculated as the PH of that rice plant using the Zonal Statistics function of ArcMap 10.8. The PH inputs corresponding to the AGB samples in the regression model were the mean values of PH extracted within the plot or field on the day of destructive sampling. In addition, 20 VI maps were also calculated from five single-band reflectance maps using the Raster Calculator function. The calculation formulas are shown in **Table 3**.

**Table 3:** Vegetation indices (VIs) used in this study.

NO.	Index	Abbreviation	Formula	Reference
1	Chlorophyll Index Green	CIg	(NIR/Green)−1	(Gitelson et al., 2003)
2	Chlorophyll Index RedEdge	CIre	(NIR/RedEdge)−1	(Gitelson et al., 2003)
3	Chlorophyll Vegetation Index	CVI	(NIR/Green)×(Red/Green)	(Vincini et al., 2008)
4	Difference Vegetation Index	DVI	NIR−Red	(Jiang et al., 2006)
5	Enhanced Vegetation Index	EVI	2.5(NIR−Red)/(NIR+6Red−7.5Blue+1)	(Huete et al., 2002)
6	Enhanced Vegetation Index 2	EVI2	2.5(NIR−Red)/(NIR+2.4Red+1)	(Jiang et al., 2008)
7	Green Leaf Index	GLI	(2Green−Red−Blue)/(2Green+Red+Blue)	(Gobron et al., 2000)
8	Green Normalized Difference Vegetation Index	GNDVI	(NIR−Green)/(NIR+Green)	(Gitelson et al., 1996)
9	Leaf Chlorophyll Index	LCI	(NIR−RedEdge)/(NIR+Red)	(Datt, 1999)
10	Modified Chlorophyll Absorption in Reflectance Index	MCARI	((RedEdge−Red)−0.2(RedEdge−Green)) ×(RedEdge/Red)	(Daughtry et al., 2000)
11	Modified Simple Ratio	MSR	$((\text{NIR}/Red)-1)/\sqrt{(\text{NIR}/Red)+1}$	(Haboudane et al., 2004)
12	Normalized Difference RedEdge	NDRE	(NIR−RedEdge)/(NIR+RedEdge)	(Barnes et al., 2000)
13	Normalized Difference Vegetation Index	NDVI	(NIR−Red)/(NIR+Red)	(Rouse et al., 1973)
14	Optimized Soil Adjusted Vegetation Index	OSAVI	(NIR−Red)/(NIR+Red+0.16)	(Rondeaux et al., 1996)
15	Renormalized Difference Vegetation Index	RDVI	$(\text{NIR}-\text{Red})/\sqrt{\text{NIR}+\text{Red}}$	(Roujean and Breon, 1995)
16	Ratio Vegetation Index	RVI	NIR/Red	(Jordan, 1969)
17	Soil Adjusted Vegetation Index	SAVI	1.5(NIR−Red)/(NIR+Red+0.5)	(Huete, 1988)
18	Triangular Vegetation Index	TVI	(120(NIR−Green)−200(Red−Green))/2	(Broge and Leblanc, 2001)
19	Visible Atmospherically Resistant Index	VARI	(Green−Red)/(Green+Red−Blue)	(Gitelson et al., 2002)
20	Wide Dynamic Range Vegetation Index	WDRVI	(0.2NIR−Red)/(0.2NIR+Red)	(Gitelson, 2013)

To facilitate the accurate extraction of rice canopy features from the images, it was essential to exclude the interstitial soil areas between adjacent rice plants. The maximum inter-class variance method (OTSU algorithm) (Otsu, 1979) was applied in MATLAB R2021b to extract the rice canopy. This algorithm returned a threshold for effectively distinguishing between foreground (rice plants) and background (soil). The segmentation threshold for plants and soil was determined using NIR band images in this study. The rice canopy extracted according to this threshold then served as a mask for extracting VI maps. In plot experiments, the outermost two rows of plants on each side of the plots were excluded and the remaining rice canopy pixel points were included in the VI calculation. Additionally, the extracted rice canopy was also used to calculate canopy cover (CC). The CC of each plot or field was the ratio of the rice canopy-covered area to the total area.

### 2.4 Data analysis and modelling

#### 2.4.1 Correlation analysis

In this study, correlation analysis was carried out between features (VIs, PH and CC) obtained from UAV images and the corresponding AGB measured on each sampling day. These comparisons were based on the Pearson correlation coefficient (*r*) (Lee Rodgers and Nicewander, 1988), a dimensionless metric that assesses the degree of correlation between variables. The value of *r* ranges from −1 to 1 and an absolute value closer to 1 indicates a stronger correlation between the two variables. The formula for calculating *r* is as in Equation (1):


(1) $$r=\frac{\sum_{i=1}^{n}\left(x_{i}-\bar{x}\right)\left(y_{i}-\bar{y}\right)}{\sqrt{\sum_{i=1}^{n}{\left(x_{i}-\bar{x}\right)}^{2}\sum {\left(y_{i}-\bar{y}\right)}^{2}}}$$

where $\bar{\text{x}}$ and $\bar{\text{y}}$ are the mean values of variables x and y, respectively.

#### 2.4.2 Modelling methods

Highly correlated data points from multiple samplings were chosen to create the model dataset. This dataset was divided randomly into two subsets: a training set, including 70% of the dataset, and a separate test set, comprising the remaining 30%. To avoid the performance of the model being affected by the order of magnitude of different features, it was essential to pre-process the data. Standardization was employed, using the mean and standard deviation, to scale and normalize the data consistently (Milligan and Cooper, 1988). The formula for standardization is as in Equation (2):


(2) $$z=\frac{x_{i}-\bar{x}}{\sigma }$$

where $\bar{\text{x}}$ and *σ* are the mean and the standard deviation of the variable x, respectively.

Five regression methods were employed in this study, including Multiple Stepwise Regression (MSR), Elastic Net Regression (ENet), Partial Least Squares Regression (PLSR), Random Forest Regression (RF) and Support Vector Regression (SVR). All model construction was completed in Python.

Multiple Stepwise Regression is a method of iteratively checking the significance of each independent variable to finally obtain the independent variable that has a significant effect on the dependent variable (Ku and Popescu, 2019). It includes three methods: forward selection, backward elimination and bidirectional elimination. In this study, forward stepwise regression was used and the adjusted R² value was selected to compare the performance of the model. The independent variables that contributed the most to the model were selected to be added. The final multivariate linear model (Altman and Krzywinski, 2015) was built using those selected independent variables.

Elastic Net Regression is a regularized regression method based on a linear model that combines the L1 and L2 penalties of the Lasso and Ridge methods (Zou and Hastie, 2005). Ridge regression retains all variables in the model and is not applicable to feature selection. Lasso regression does not solve the problem of rational selection among highly correlated features. ENet as a combination of both has advantages in dealing with multicollinearity as well as reducing overfitting. In this study, the strength and scale of regularization were determined based on the parametric grid search.

Partial Least Squares Regression, as a widely used algorithm, can address the problem of covariance between independent variables and enables dimensionality reduction in the latent variable space (Mashimbye et al., 2012). The number of features to be retained after dimensionality reduction can be specified. It can explain as much as possible about the relationship between the original independent variables and the dependent variable providing strong model interpretability. In this study, the optimal number of features after dimensionality reduction was found by loop traversal for different combinations of feature inputs.

Random Forest Regression is an ensemble learning method that operates by constructing a large number of decision trees while training (Izquierdo-Verdiguier and Zurita-Milla, 2020). RF avoids the problem of overfitting in the Decision tree model and it returns the average prediction of individual trees in a regression task. In this study, the number of decision trees, maximum depth, and maximum number of randomly selected features for each decision tree were determined by parametric grid search. Importance scores of features in the RF algorithm were used to support feature selection.

Support vector regression constructs a regression model by finding a set of data points that are closest to the target value in the feature space, which is called a support vector, to fit the data (Smola and Schölkopf, 2004). The goal of SVR is to find the best hyperplane at the maximum interval to minimize the total prediction error. In this study, the type of kernel, regularization parameter, and kernel coefficients used in support vector machines were determined by parametric grid search.

To explore what combination of spectral features, spatial parameters and the temporal parameter can achieve the optimal AGB estimation results, six feature combination scenarios were considered for each method: (1) VIs; (2) PH and CC; (3) VIs, PH, and CC; (4) VIs and DAT; (5) PH, CC, and DAT; (6) VIs, PH, CC, and DAT. Grid search was employed to find a more optimized group of parameters.

#### 2.4.3 Model accuracy assessment

The models built by each method on the training set were cross-validated with four folds. The K-fold cross-validation is a common method of assessing model performance (Zhang and Liu, 2023). It divides the dataset into K folds and trains the model K times. A different fold is used as the validation set each time and the remaining folds are used as the training set. The results of each training are averaged to obtain the final model performance estimate. The cross-validation can effectively reduce the model overfitting problem and improve the reliability and generalization performance (Vu et al., 2022).

To evaluate the accuracy of the extracted Plant Height (PH) and the performance of the regression models, two metrics were employed: the coefficient of determination (*R^2^*) (Karch, 2020), the root mean square error (*RMSE*) (Hyndman and Koehler, 2006) and the normalized root mean square error (*NRMSE*) (Khan and Osińska, 2023). *R^2^* serves as an indicator of the proportion of variance explained by the model relative to the total variance and typically falls between 0 and 1. Higher *R^2^* values signify better model explanatory power, with values closer to 1 indicating a stronger fit.

*RMSE* is a widely used statistic for quantifying the discrepancy between estimated and actual values. It’s particularly suitable for comparing different methods on the same dataset since it usually has the same units as the estimated and true values. *NRMSE* is the normalized value of *RMSE*, usually expressed as a percentage. Smaller values of *RMSE* and *NRMSE* indicate a closer correspondence between estimated and actual values, reflecting a superior fitting performance of the model. Conversely, larger *RMSE* and *NRMSE* values suggest diminished model accuracy.

The testing results of the AGB estimation model on field samples were assessed using percent error (%error). The formulas for calculating *R^2^*, *RMSE*, *NRMSE* and %error are as in Equations (3–6):


(3) $$R^{2}=1-\frac{\sum_{i=1}^{n}{\left(x_{i}-y_{i}\right)}^{2}}{\sum_{i=1}^{n}{\left(x_{i}-\bar{x}\right)}^{2}}$$

(4) $$RMSE=\sqrt{\frac{1}{n}\sum_{i=1}^{n}{\left(y_{i}-x_{i}\right)}^{2}}$$

(5) $$NRMSE=\frac{RMSE}{x_{max}-x_{min}}$$

(6) $$\%\text{error}=\frac{\left|y_{i}-x_{i}\right|}{x_{i}}\times 100\%$$

where n is the number of data points, $x_{i}$ is the true value of the data point, *y_i_* is the estimated value of the data point, $\bar{x}$ , $x_{max}$ and *x_min_* are the mean, maximum and minimum values of true values, respectively.

#### 2.4.4 Model interpretation

In addition to focusing on model accuracy, the correct interpretation of model outputs is also significant for model improvement. Simple models (e.g., linear models) are easy to interpret but may be less accurate. Complex models are usually more accurate but, as a ‘black box’, often have complicated internal mechanisms that are difficult to explain in concrete ways. For this reason, Shapley additive explanations (SHAP) (Lundberg and Lee, 2017) was used in this study to interpret the predictions of each regression model as a unified measure of the feature importance. The concept of Shapley values was derived from the theory of cooperative games and can be used to measure the marginal contribution of each feature to the final output. SHAP analysis does not depend on the internal mechanism of the model, so it is suitable for various types of models with inputs and outputs, such as linear regression models, tree-based models and neural networks.

In this study, the Shapley value calculation was implemented with the help of a module package in Python. The results were visualized through bee swarm plots, with features ordered by their importance level from top to bottom. Positive Shapley values for each feature signified a positive impact on increasing the predicted value, while negative values indicated a negative impact. Furthermore, the color of the dots represented the specific feature value.

## 3 Results

### 3.1 Comparison between measured and extracted PH

**Figure 3:**
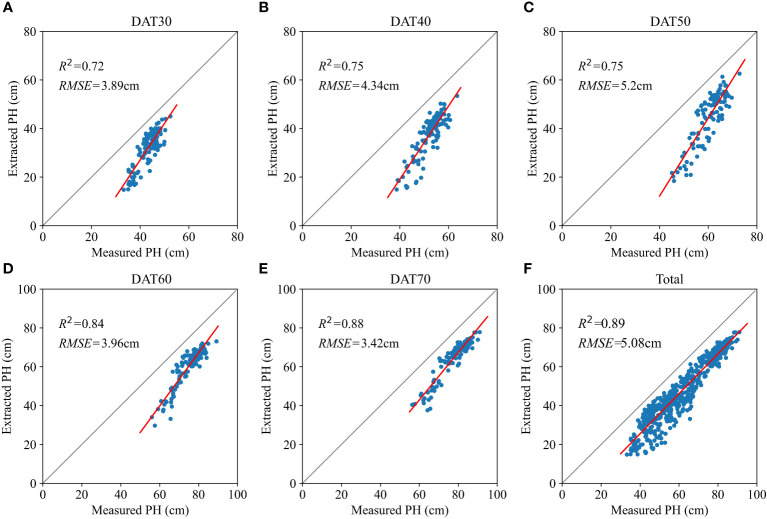
Correlation between the ground-measured plant height (PH) and extracted PH from UAV images from tillering to milk ripening stages: **(A, B)** tillering stage; **(C)** jointing-booting stage; **(D)** heading-flowering stage; **(E)** milk ripening stage; **(F)** total.

The comparison between ground-measured PH and PH extracted from the DSM was conducted from tillering to milk ripening stages (DAT30, DAT40, DAT50, DAT60 and DAT70) in the year 2022, as illustrated in **Figure 3**. Each measurement involved a total of 120 observation plants distributed across 20 plots. **Figure 3** demonstrates a strong correlation between ground-measured PH and extracted PH. Notably, as the rice plants matured, this correlation exhibited a manifest strengthening trend.

In the first three measurements, the *R^2^* values were 0.72, 0.75 and 0.75, and the *RMSE* values were 3.89, 4.34 and 5.2 cm, respectively. Remarkably, for the latter two measurements, the *R^2^* values were 0.84 and 0.88, and the *RMSE* values were 3.96 and 3.42 cm, respectively, demonstrating higher correlations. When considering all five measurements collectively, the overall *R^2^* value reached 0.89 and the *RMSE* was 5.08 cm, signifying that the utilization of 3D point cloud data obtained from UAV facilitated a convenient and relatively accurate measurement of rice PH.

### 3.2 Correlation analysis between features and AGB at different growth stages of rice

**Figure 4:**
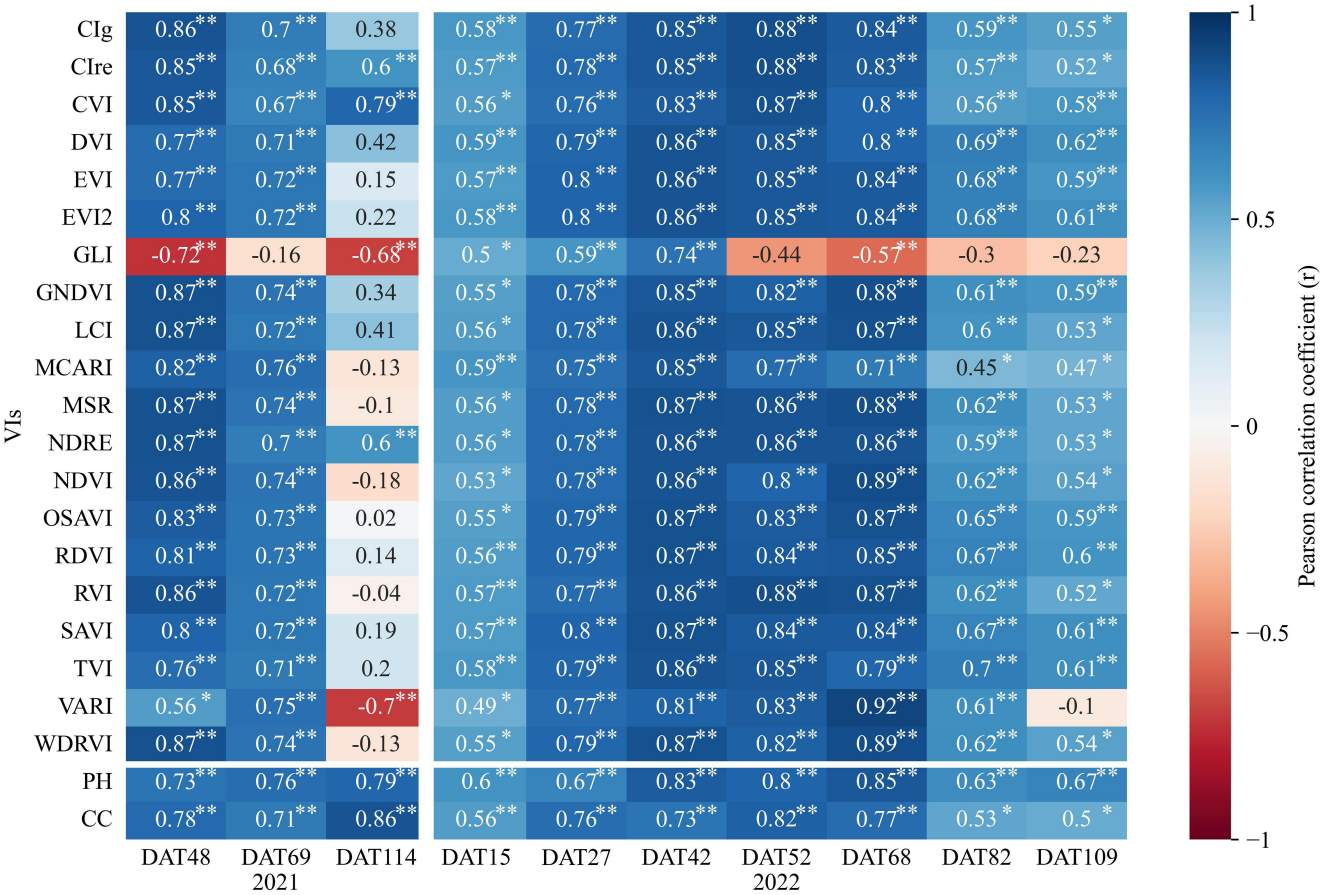
Pearson correlation coefficient (*r*) between 20 vegetation indices (VIs), plant height (PH), canopy cover (CC) with above-ground biomass (AGB) at different growth stages of rice. * and ** indicate significance at the 0.05 and 0.01 significance levels, respectively.

The study examined the correlation between the 20 VIs, PH, CC extracted from UAV images and AGB of rice plants in 2021 and 2022, as illustrated in **Figure 4**. Overall, correlations between these variables followed a pattern of increasing and then decreasing as the rice grew. Notably, during the mid-tillering to flowering stages (DAT48 and DAT69 in 2021, DAT27, DAT42, DAT52 and DAT68 in 2022), most VIs showed strong correlations with AGB (p< 0.01). In 2021, VIs like GNDVI, LCI, MSR, NDRE and WDRVI reached correlations as high as 0.87 at DAT48. In 2022, VARI had the highest correlation with AGB at DAT68, reaching 0.92. However, during early and late growth stages (DAT114 in 2021, DAT15, DAT82 and DAT109 in 2022), the correlations were weaker or non-existent. PH and CC also showed significant correlations with AGB, suggesting their potential for improving AGB estimation accuracy based on VIs.

### 3.3 Regression modelling for AGB

**Figure 5:**
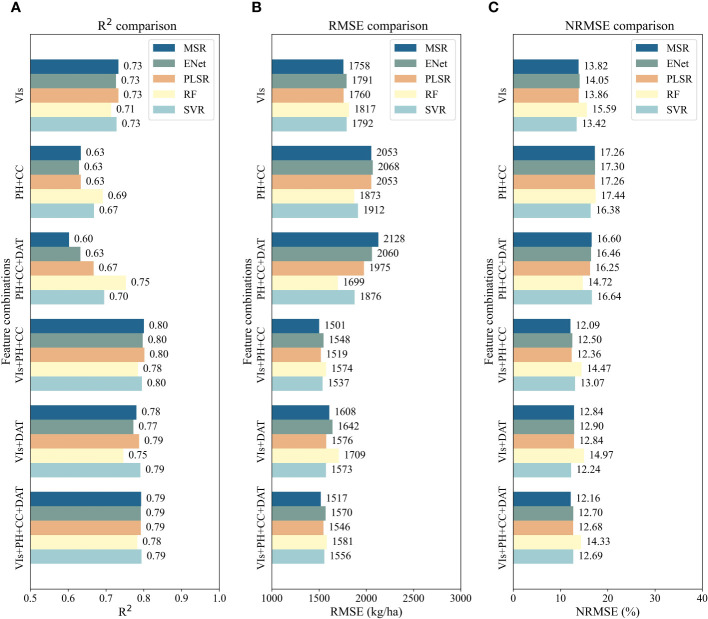
Average accuracy of four-fold cross-validation of the AGB estimation models built from five regression algorithms (MSR, ENet, PLSR, RF and SVR), each using six feature combinations (VIs, PH+CC, VIs+PH+CC, VIs+DAT, PH+CC+DAT, VIs+PH+CC+DAT): **(A)** R^2^ comparison; **(B)** RMSE comparison; **(C)** NRMSE comparison.

Based on the results in section 3.2, a total of 120 groups of data from the six samplings (DAT48 and DAT69 in 2021, DAT27, DAT42, DAT52 and DAT68 in 2022) were collected as a plot dataset to estimate the rice AGB during the major growth stages (from mid-tillering to flowering stages). The training set consisted of 84 groups randomly selected from the dataset. The test set consisted of the remaining 36 groups. The mean results of the four-fold cross-validation on the training set of five regression models are shown in **Figure 5**, each employing six different combinations of features. When only VIs were used as the independent variable, the MSR and PLSR models had similar accuracy results with *R^2^* values of 0.73 and *RMSE* values of 1758 and 1760 kg/ha, respectively. Models constructed from spatial parameters (PH and CC) were all less accurate. Among them, the RF model performed the best (*R^2^ =* 0.69, *RMSE*=1873 kg/ha). With either VIs as features or PH and CC as features, the mean *NRMSE* of the four-fold cross-validation of the SVR model was the lowest compared to the other models, namely 13.42% and 16.38%, respectively. With the addition of the temporal parameter (DAT) to the spatial parameters, the accuracy of the other four models, except for the MSR model, improved. In particular, the *R^2^* value of the RF model increased to 0.75 and the *RMSE* value decreased to 1699 kg/ha.

As shown in **Figure 5**, the addition of spatial parameters to the VIs together as input features can significantly improve the estimation accuracy of rice AGB. When VIs, PH and CC were used as model inputs, the MSR and PLSR models had *R^2^* values that exceeded 0.80, *RMSE* values of 1501 and 1519 kg/ha, and *NRMSE* values of 12.09% and 12.36%, followed closely by the ENet, SVR and RF models. Similarly, the combination of VIs and DAT provides better estimation accuracy for AGB than the models constructed from VIs only, but slightly lower than those constructed from VIs, PH and CC. When VIs, PH, CC, and DAT were all taken as the features, the estimation accuracy of all five regression models was also good. The MSR, ENet, PLSR and SVR models obtained quite similar results in terms of *R^2^* values, with the MSR model having the lowest *RMSE* value of 1517 kg/ha and *NRMSE* value of 12.16%. Overall, it was shown that the feature combination of VIs with spatial parameters was superior to that of VIs with spatio-temporal parameters, followed by that of VIs with temporal parameters.

### 3.4 Importance analysis of model features

**Figure 6:**
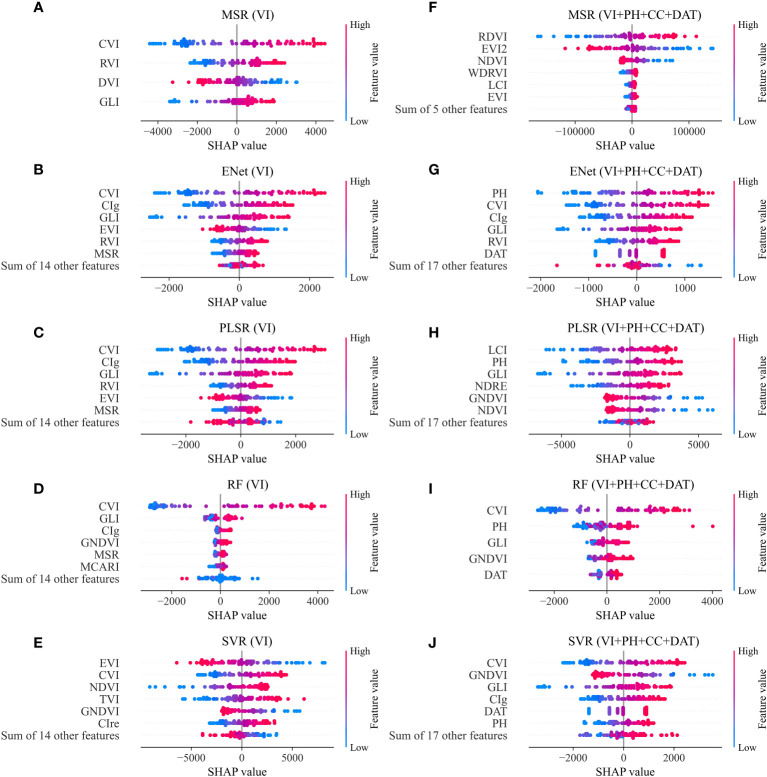
Importance ranking of features in different AGB estimation models constructed from two types of feature inputs: **(A–E)** VIs; **(F–J)** VIs, PH, CC and DAT.

**Figure 6** illustrates the feature importance of the AGB estimation models constructed from VIs and combinations of VIs, PH, CC and DAT. In the models built with VIs only, the index CVI made the highest contribution to the MSR, ENet, PLSR and RF model outputs, and the second highest contribution to the SVR model output. Moreover, other indices that contributed more to the output in several models included RVI, GLI, CIg, EVI and MSR.

When VIs, PH, CC and DAT were all applied as model input, the PH made a significant contribution to the results of the models. In the ENet model, it was the feature PH that contributed the most to the output, and PH ranked second in the PLSR and RF model. The CVI index also contributed greatly to the ENet, RF and SVR model outputs. In addition, the VIs that contributed significantly to the output of each model included RDVI, EVI2 and NDVI in the MSR model, GLI in the ENet, PLSR, RF and SVR model, LCI in the MSR and PLSR model, GNDVI in the PLSR, RF and SVR model.

Furthermore, as can be seen from the distribution of feature value in **Figure 6**, the sample points with high feature values of VIs such as RDVI, CVI, CIg and LCI have mostly positive SHAP values, while the sample points with low feature values of these VIs have mostly negative SHAP values. This indicated that when the value of these VIs was higher, the estimated AGB value would be larger. On the contrary, the lower their value, the smaller the estimated AGB value would be. Thus, the DVI and EVI2 in the MSR model and GNDVI in the PLSR and SVR model showed the opposite property. Their larger feature values decreased the estimated AGB while smaller feature values increased the estimated AGB.

### 3.5 Testing results of AGB estimation models on experimental plot data

**Figure 7:**
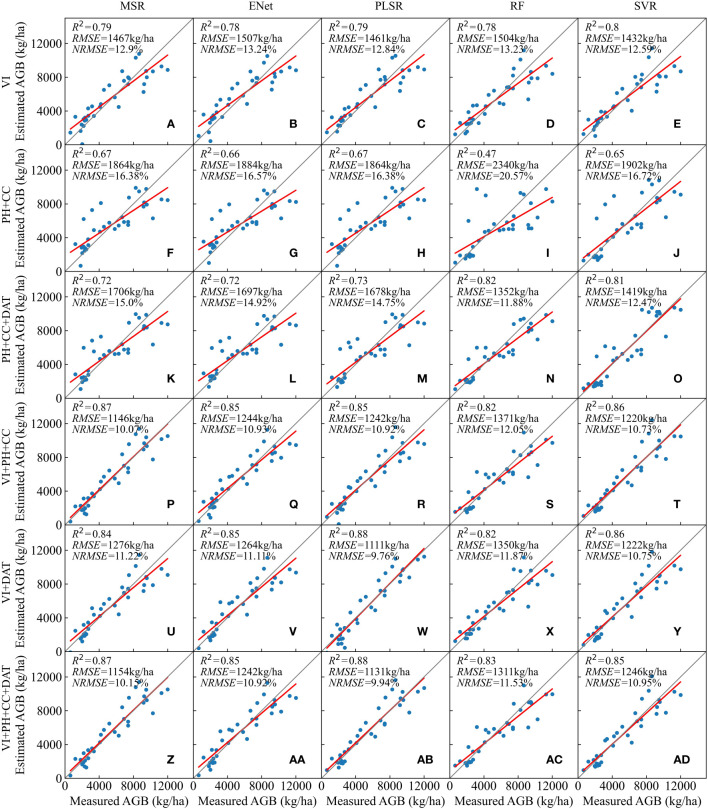
Testing results of the AGB estimation models on the test set of plot dataset: **(A–E)** VIs; **(F-J)** PH+CC; **(K-O)** PH+CC+DAT; **(P-T)** VIs+PH+CC; **(U-Y)** VIs+DAT; **(Z-AD)** VIs+PH+CC+DAT.

The testing results of the established regression models on the independent test set of the plot dataset are shown in **Figure 7**. Among the models using VIs as the independent variables, the SVR model had the highest accuracy with an *R^2^* value of 0.8, an *RMSE* value of 1432 kg/ha, and an *NRMSE* value of 12.59%. In addition, the *R^2^* value of the other four models all exceeded 0.78. AGB models constructed from the spatial parameters PH and CC all had relatively lower testing accuracy with their *R^2^* values ranging from 0.47 to 0.67 and *RMSE* values ranging from 1864 to 2340 kg/ha. Also, the addition of the temporal parameter DAT based on PH and CC could improve the testing accuracy of AGB to a certain extent.

Models constructed from combinations of VIs along with PH and CC all achieved relatively high testing results. Among them, the MSR model showed the best performance on the test set with an *R^2^* value of 0.87, an *RMSE* value of 1146 kg/ha, and an *NRMSE* value of 10.07%, followed by the SVR, ENet and PLSR models. In the case of VIs and DAT as features, the PLSR model showed the most accurate result with an *R^2^* value as high as 0.88, an *RMSE* value of 1111 kg/ha, and an *NRMSE* value of 9.76%. Furthermore, with all parameters included as the independent variable, again the PLSR model showed the best accuracy (*R^2^ =* 0.88, *RMSE*=1131 kg/ha, *NRMSE*=9.94%).

### 3.6 Testing results of AGB estimation models on sampling field data

To explore the applicability of the established AGB estimation models on large-scale fields, the models were tested on five extra fields and the percent error between their estimated and measured values was calculated. The input features considered included VIs and their combinations with spatial and temporal features. The estimation results of different models and their errors are shown in **Table 4**.

**Table 4:** Testing results of AGB estimation models on large-scale field data.

Input features	Field	Methods									
		MSR		ENet		PLSR		RF		SVR	
		Estimated AGB (g/plant)	%Error	Estimated AGB (g/plant)	%Error	Estimated AGB (g/plant)	%Error	Estimated AGB (g/plant)	%Error	Estimated AGB (g/plant)	%Error
VIs	F1	161.8	65.0%	98.7	0.6%	108.7	10.9%	29.2	70.2%	216.0	120.3%
	F2	149.9	54.1%	88.6	9.0%	95.5	1.8%	27.9	71.3%	221.3	127.5%
	F3	152.2	49.8%	87.8	13.5%	92.3	9.1%	27.9	72.5%	258.7	154.7%
	F4	165.6	55.6%	101.2	4.9%	112.4	5.6%	29.3	72.5%	212.2	99.4%
	F5	187.1	59.1%	118.9	1.1%	137.7	17.1%	29.7	74.8%	169.2	43.9%
VIs+PH+CC	F1	40.6	58.6%	93.6	4.5%	113.7	16.0%	36.2	63.1%	166.1	69.4%
	F2	48.3	50.3%	92.4	5.0%	111.9	15.1%	34.6	64.4%	168.1	72.8%
	F3	41.6	59.1%	91.1	10.3%	110.5	8.8%	34.6	65.9%	180.5	77.7%
	F4	42.7	59.9%	97.8	8.1%	117.9	10.7%	36.6	65.6%	169.8	59.6%
	F5	42.6	63.8%	112.8	4.0%	135.8	15.5%	37.1	68.4%	183.9	56.4%
VIs+DAT	F1	120.7	23.1%	97.3	0.7%	123.1	25.5%	28.7	70.7%	123.8	26.2%
	F2	119.0	22.3%	88.9	8.6%	118.1	21.5%	28.2	71.0%	114.8	18.1%
	F3	116.5	14.7%	90.1	11.3%	126.6	24.7%	28.2	72.2%	119.3	17.5%
	F4	120.0	12.7%	99.7	6.4%	125.1	17.6%	28.9	72.8%	126.4	18.8%
	F5	103.1	12.3%	115.6	1.7%	123.9	5.4%	29.0	75.4%	140.5	19.4%
VIs+PH+CC +DAT	F1	39.6	59.6%	94.5	3.6%	103.4	5.5%	36.3	63.0%	127.8	30.3%
	F2	47.6	51.1%	91.3	6.1%	105.9	8.9%	34.6	64.4%	123.3	26.8%
	F3	40.7	59.9%	91.2	10.2%	106.4	4.8%	34.6	65.9%	126.5	24.5%
	F4	41.8	60.7%	97.4	8.5%	108.9	2.3%	36.2	66.0%	129.9	22.0%
	F5	41.8	64.4%	113.5	3.5%	132.8	12.9%	37.4	68.2%	140.8	19.7%

Considering four different feature input situations, the estimation results of the ENet model were optimal on all five fields. When using VIs and DAT as inputs, the ENet model had an average %error of only 5.7% in estimating AGB for the five fields, followed by VIs as inputs (5.8%), VIs, PH and CC as inputs (6.4%), VIs, PH, CC and DAT as inputs (6.4%). For the PLSR method, with all of VIs, PH, CC and DAT as inputs, the best AGB estimation results could be obtained (6.9%). Compared to the other three feature combinations, the MSR and SVR models showed relatively better results when VIs and DAT were used as inputs, with average %error of 17.0% and 20%, respectively. Notably, when the stepwise regression method selected a large number of parameters, it may lead to the constructed linear model having a relatively large error on the new data. For all five fields, the RF model combined with different feature combinations produced results with large errors, with %error ranging from 63% to 75.4%. Overall, the ENet and PLSR models performed better for AGB estimation on large fields compared to the other models. Moreover, the introduction of temporal or spatial parameters can improve the accuracy of these models in estimating AGB in large fields to some extent.

## 4 Discussion

### 4.1 Performance evaluation of different regression models for estimating AGB

Five regression methods, MSR, ENet, PLSR, RF and SVR, were used in this study to construct the AGB estimation model. In terms of the results of the models on the test set of the plot dataset, the performance of the five models varied when facing high-dimensional data (**Figure 7**). For the MSR method, the introduction of the DAT feature into the model characterized by VIs, PH and CC did not result in any further improvement in model testing accuracy. Similarly, for the SVR method, the introduction of extra parameters into the model characterized by VIs and DAT or VIs, PH and CC did not result in any improvement in the testing accuracy. This may be attributed to the overfitting caused by a larger number of features (Sim et al., 2018). In comparison, the RF model performed better (Zhang et al., 2022). In this study, feature selection was carried out based on the importance scores of features in the RF algorithm (Li et al., 2017). Whether the DAT feature was introduced over VIs, PH, and CC, or PH and CC over VIs and DAT, the RF model demonstrated a noticeable improvement in the testing accuracy.

In addition, we found that the RF model performs better with high-dimensional data than with low-dimensional data. When two feature combinations (PH+CC, PH+CC+DAT) were used as model inputs, the training results of the RF model were both optimal, with *R^2^* values of 0.69 and 0.75, and *RMSE* values of 1873 and 1699 kg/ha, respectively. However, the accuracy of the RF model on the test set was the lowest when PH and CC were used as features, with an *R^2^* value of only 0.47 and an *RMSE* value exceeding 2000 kg/ha. In contrast, the testing accuracy remained high when PH, CC, and DAT were used as features.

The MSR, ENet, and PLSR methods are all linear regression methods. When VIs, PH, CC and DAT were all included in the model features, the ENet model had the lowest accuracy. This may be attributed to the possibility that although the ENet model combines the characteristics of Lasso and Ridge regression, it does not eliminate the shortcomings in handling multicollinearity and feature selection. Therefore, some bias and inconsistency in parameter estimation and the problem of high variance in Lasso estimates remain (Kayanan and Wijekoon, 2019, 2020). In dealing with multicollinearity and feature selection, the MSR model can obtain a reliable and effective set of feature combinations through stepwise selection (Kolasa-Wiecek, 2015), while the PLSR model can control the complexity of the model by setting the number of Latent Variables to achieve dimensionality reduction (Shen et al., 2020). In this study, the more appropriate process of dimensionality reduction allowed the MSR and PLSR models to outperform the ENet model on the test set.

### 4.2 Combining spatial and temporal parameters to improve the AGB estimation accuracy

In this study, we investigated the accuracy differences of AGB regression models for various combinations of spectral features (VIs), spatial parameters (PH and CC), and the temporal parameter (DAT). As can be seen from **Figures 5** and **7**, compared with models constructed from spectral features or spatial parameters alone, the combination of these features showed a significant improvement in estimation accuracy. The increase in rice PH in the vertical direction and the expansion of CC in the horizontal direction were generally consistent with the continuous increase of rice AGB. Therefore, the combination of PH and CC can visualize the changes in rice AGB in a 3D space (Maimaitijiang et al., 2019). Similar studies (Adeluyi et al., 2022; Shu et al., 2023) have also suggested the positive significance of PH and CC for AGB estimation, which is consistent with the findings of this study.

Image resolution affects the accuracy of AGB estimation using spectral information and crop surface models (Modica et al., 2020; Liu et al., 2021). Low spatial resolution reduces image quality. High spatial resolution brings more details of the crop canopy but captures environmental noise from soil, weeds, and shadows at the same time (Liu et al., 2022b; Zhu et al., 2023). Considering the large areas of the sampling fields, we did not employ the same flight height of image acquisition as the experimental plots in terms of practicality. The five groups of field samples were mainly used to test the applicability of the models. Although the spatial resolution of the large sampling fields (1.9 cm/px) was slightly lower than that of the experimental plots (0.5 cm/px), the ENet and PLSR models still achieved good estimation results in this study. We will consider constructing estimation models with greater applicability across field data in the future. In addition, the impact of extracted PH tending to be lower than the actual PH values needs to be further investigated (Willkomm et al., 2016; Ji et al., 2022). Here, the underestimation of PH may be attributed to the sparse canopy point cloud, which could be occasionally mistaken for the soil point cloud when the rice canopy cover was minor. We will further calibrate the extracted PH values in subsequent studies to match the actual conditions more closely and thus improve the accuracy. In addition, the CC in this study was calculated by extracting the rice canopy using only one algorithm, OTSU. Combining methods such as machine learning may improve the accuracy of CC and further enhance the accuracy of AGB estimation.

From a temporal perspective, the growth of rice biomass conforms to the rule of the Logistic curve, which can be characterized as slow growth at the initial stage, rapid growth in the middle stage, slow growth in the later stage, and eventually converging to the maximum value infinitely (Yu et al., 2002). Therefore, the growing days of rice can also function as a parameter for estimating AGB and can compensate for the possible saturation of VIs, PH, and CC in the later stages of rice growth. In this study, the results show that introducing the temporal parameter DAT based on VIs can indeed improve the estimation accuracy of AGB. In particular, when the spatial parameters of rice cannot be obtained due to objective conditions, adding DAT to spectral features to estimate AGB is also an alternative. On the whole, the feature combinations of VIs, PH, CC, and DAT showed superior evaluation performance for all five modelling approaches (**Figure 7**). For the MSR and ENet models, the combination of VIs, PH, CC, and DAT demonstrated similar test accuracy to the combination of VIs, PH, and CC; for the PLSR model, the combination of VIs, PH, CC, and DAT demonstrated similar test accuracy to the combination of VIs and DAT.

Overall, we took advantage of the convenience of obtaining, processing and analyzing point cloud data and multispectral remote sensing images. Considering that the information on multispectral bands is limited and sensitive to the atmosphere and clouds, integrating multi-source remote sensing data is one future study target (Yu et al., 2023; Zhai et al., 2023; Liu et al., 2023b). For instance, hyperspectral remote sensing, synthetic aperture radar (SAR), and thermal infrared remote sensing (TIR) could provide more abundant spectral and temperature information, and help to minimize the influence of weather and environmental factors (Alebele et al., 2020; Liu et al., 2022a; Xu et al., 2023; Zhang et al., 2024). Spectral analysis methods such as wavelet analysis could also be employed to provide more integrated and in-depth data (Wang et al., 2022b).

### 4.3 Generalization ability of regression algorithms on different nitrogen samples

**Figure 8:**
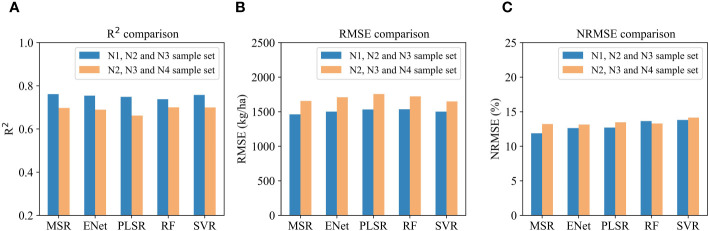
Accuracy of different AGB estimation models trained from the N1, N2 and N3 sample set and the N2, N3 and N4 sample set: **(A)** R^2^ comparison; **(B)** RMSE comparison; **(C)** NRMSE comparison.

To explore the generalization ability of each regression algorithm, samples with low-nitrogen (N1) and high-nitrogen (N4) treatments were used for testing, respectively and the remaining treatments were used as the training set. Thus, two scenarios were included, one using the N1, N2, and N3 sample set to train the model and test it on the N4 sample, and the other using the N2, N3, and N4 sample set to train the model and test it on the N1 sample. VIs, PH, CC and DAT were all taken as model features. The constructed models were also cross-validated with four folds and the average accuracy results are shown in **Figure 8**. Among the models trained from the N1, N2 and N3 sample set, the MSR model had the highest *R^2^* value of 0.76 and the lowest *RMSE* value of 1461 kg/ha, followed by the SVR, ENet and PLSR models. As for the models trained from the N2, N3 and N4 sample set, the RF, SVR and MSR models had similar accuracy, with their *R^2^* values exceeding 0.69. Overall, the accuracy of the AGB estimation models constructed from the N1, N2, and N3 sample set was higher than that of models constructed from the N2, N3, and N4 sample set.

**Figure 9:**
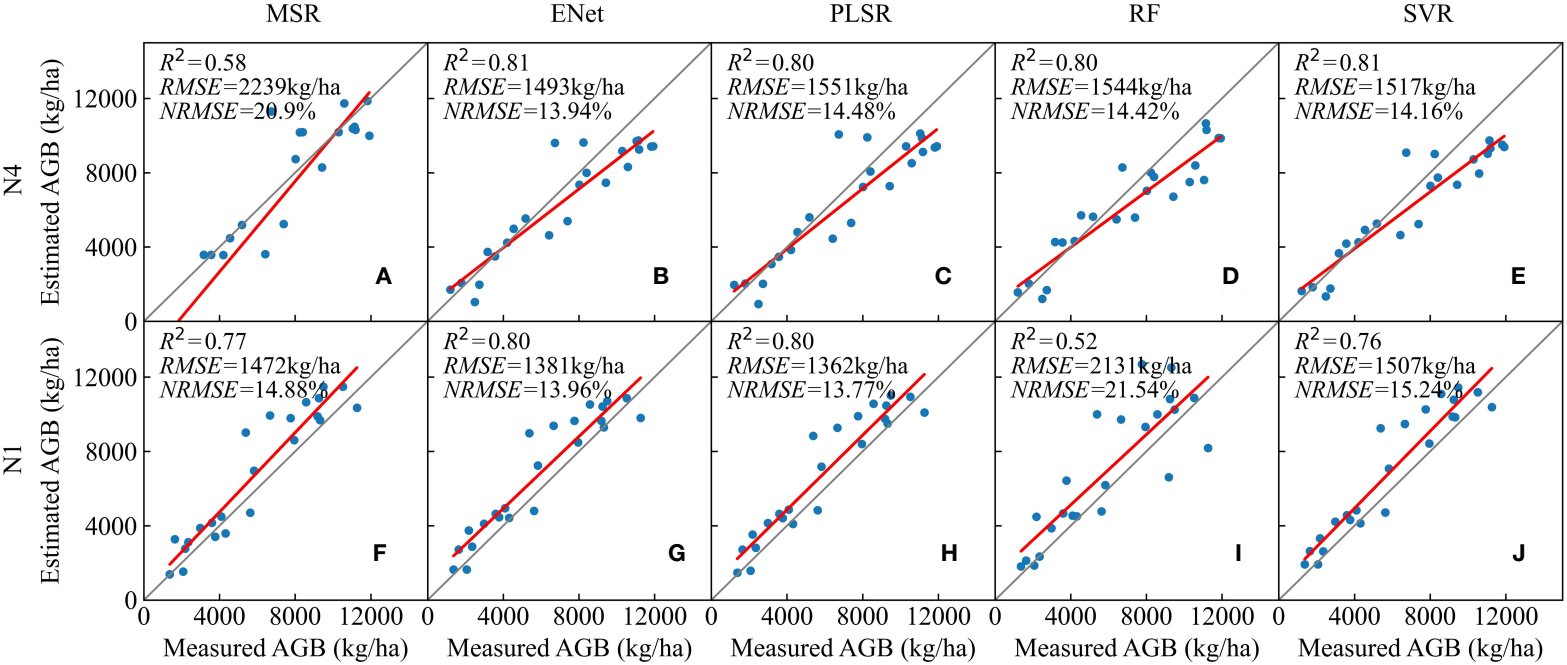
Testing results of different AGB estimation models trained from the N1, N2 and N3 sample set on the highest nitrogen treatment (N4) sample **(A–E)** and models trained from the N2, N3 and N4 sample set on the lowest nitrogen treatment (N1) sample **(F–J)**.

The testing results of the models for the two scenarios above are shown in **Figure 9**. The estimated results for the majority of the N4 sample points were smaller than the measured values, while those for the majority of the N1 sample points were larger than the measured values. The better models for estimating the N4 treatment were the ENet and SVR models with their *R^2^* values up to 0.81. The PLSR and RF models had similar estimation accuracy, with *R^2^* values of 0.80. In addition, the best model for estimating N1 treatment was the PLSR model with an *R^2^* value of 0.80, an *RMSE* value of 1362kg/ha and an *NRMSE* value of 13.77%. The RF model had the lowest estimation accuracy for the N1 sample, with an *R^2^* value of only 0.52. Although the accuracy of the modelling set performed well, the MSR model obtained the lowest accuracy in predicting the N4 samples. This may be attributed to overfitting. Studies have shown that small-scale training may cause the problem of overfitting (Dai et al., 2022). For a small training set consisting only some of the samples, MSR selected a relatively large number of features, resulting in a good performance on the training set and a poor adaptation on unseen data. A similar result was observed when the RF model predicted N1 samples. Therefore, a large and representative amount of training data is crucial to achieve a good generalization ability of the constructed model, especially when employing machine learning algorithms (Xin et al., 2020; Cao et al., 2021). In general, the ENet and PLSR models had better generalization performance for new data from both high-nitrogen and low-nitrogen samples in this study.

## 5 Conclusions

To further enhance the accuracy of estimating rice AGB through VIs, this study proposed an innovative multidimensional estimation method that comprehensively considers spectral features, spatial structure, and phenological stages. We employed UAV-borne visible and multispectral cameras to capture digital surface models and canopy multispectral images of rice at various phenological stages. From these, we accurately extracted 20 VIs, PH, and CC. The AGB estimation models across phenological stages were constructed using five regression methods (MSR, ENet, PLSR, RF and SVR) with six different feature combinations (VIs alone, PH+CC, PH+CC+DAT, VIs+PH+CC, VIs+DAT, VIs+PH+CC+DAT). Effective testing was conducted on both plot and field experiments. The findings demonstrate the potential of drone technology for rapid and relatively accurate estimation of rice PH. Notably, the introduction of spatial parameters (PH and CC) and the temporal parameter (DAT) significantly improved the accuracy of AGB estimation. Among the models, the PLSR model with VIs and DAT as inputs, or with VIs, PH, CC, and DAT as inputs, achieved the optimal AGB estimation on plot data. Meanwhile, the ENet model showed the best estimation results on field data, highlighting its strong practical applicability. In summary, the integration of UAV remote sensing and multi-feature fusion provides an effective way to accurately estimate rice AGB. This will not only provide dependable technical guidance for remote sensing monitoring and field management of rice but also contribute to promoting the advancement of large-scale smart agriculture and precision farming.

## Data availability statement

The original contributions presented in the study are included in the article/supplementary material. Further inquiries can be directed to the corresponding authors.

## Author contributions

YD: Conceptualization, Data curation, Methodology, Visualization, Writing – original draft. SY: Project administration, Writing – review & editing, Funding acquisition. TM: Conceptualization, Funding acquisition, Methodology, Project administration, Writing – review & editing. JD: Writing – review & editing, Funding acquisition, Supervision. KC: Writing – review & editing, Investigation. GZ: Data curation, Writing – original draft. AX: Validation, Writing – review & editing. PH: Visualization, Writing – original draft. SP: Visualization, Writing – original draft. MZ: Investigation, Project administration, Writing – review & editing.

## References

[B1] Adeluyi O. Harris A. Foster T. Clay G. D. (2022). Exploiting centimetre resolution of drone-mounted sensors for estimating mid-late season above ground biomass in rice. Eur. J. Agron. 132, 126411. doi: 10.1016/j.eja.2021.126411

[B2] Alebele Y. Zhang X. Wang W. Yang G. Yao X. Zheng H.. (2020). Estimation of canopy biomass components in paddy rice from combined optical and SAR data using multi-target gaussian regressor stacking. Remote Sens. 12, 2564. doi: 10.3390/rs12162564

[B3] Altman N. Krzywinski M. (2015). Simple linear regression. Nat. Methods 12, 999–1000. doi: 10.1038/nmeth.3627 26824102

[B4] Bannari A. Morin D. Bonn F. Huete A. R. (1995). A review of vegetation indices. Remote Sens. Rev. 13, 95–120. doi: 10.1080/02757259509532298

[B5] Barnes E. M. Clarke T. R. Richards S. E. Colaizzi P. D. Thompson T. (2000). “Coincident detection of crop water stress, nitrogen status, and canopy density using ground based multispectral data,” in Proceedings of the 5th International Conference on Precision Agriculture and other resource management, Bloomington, MN USA, (Madison, WI: Precision Agriculture Center, University of Minnesota, ASA-CSSA-SSSA). July 16-19, 2000.

[B6] Bascon M. V. Nakata T. Shibata S. Takata I. Kobayashi N. Kato Y.. (2022). Estimating yield-related traits using UAV-derived multispectral images to improve rice grain yield prediction. Agriculture 12, 1141. doi: 10.3390/agriculture12081141

[B7] Bendig J. Yu K. Aasen H. Bolten A. Bennertz S. Broscheit J.. (2015). Combining UAV-based plant height from crop surface models, visible, and near infrared vegetation indices for biomass monitoring in barley. Int. J. Appl. Earth Obs. Geoinformation 39, 79–87. doi: 10.1016/j.jag.2015.02.012

[B8] Borra-Serrano I. De Swaef T. Muylle H. Nuyttens D. Vangeyte J. Mertens K.. (2019). Canopy height measurements and non-destructive biomass estimation of Lolium perenne swards using UAV imagery. Grass Forage Sci. 74, 356–369. doi: 10.1111/gfs.12439

[B9] Broge N. H. Leblanc E. (2001). Comparing prediction power and stability of broadband and hyperspectral vegetation indices for estimation of green leaf area index and canopy chlorophyll density. Remote Sens. Environ. 76, 156–172. doi: 10.1016/S0034-4257(00)00197-8

[B10] Brovkina O. Novotny J. Cienciala E. Zemek F. Russ R. (2017). Mapping forest aboveground biomass using airborne hyperspectral and LiDAR data in the mountainous conditions of Central Europe. Ecol. Eng. 100, 219–230. doi: 10.1016/j.ecoleng.2016.12.004

[B11] Cao C. Wang T. Gao M. Li Y. Li D. Zhang H. (2021). Hyperspectral inversion of nitrogen content in maize leaves based on different dimensionality reduction algorithms. Comput. Electron. Agric. 190, 106461. doi: 10.1016/j.compag.2021.106461

[B12] Chakraborty M. Panigrahy S. Sharma S. A. (1997). Discrimination of rice crop grown under different cultural practices using temporal ERS-1 synthetic aperture radar data. ISPRS J. Photogramm. Remote Sens. 52, 183–191. doi: 10.1016/S0924-2716(97)00009-9

[B13] Cheng T. Song R. Li D. Zhou K. Zheng H. Yao X.. (2017). Spectroscopic estimation of biomass in canopy components of paddy rice using dry matter and chlorophyll indices. Remote Sens. 9, 319. doi: 10.3390/rs9040319

[B14] Chin K. M. Leohken A. Sozzi D. Williams R. J. (1991). A modified Zadoks decimal code for the growth stages of rice. Trop. Pest Manage. 37, 277–280. doi: 10.1080/09670879109371599

[B15] Choi W.-J. Lee M.-S. Choi J.-E. Yoon S. Kim H.-Y. (2013). How do weather extremes affect rice productivity in a changing climate? An answer to episodic lack of sunshine. Glob. Change Biol. 19, 1300–1310. doi: 10.1111/gcb.12110 23504904

[B16] Confalonieri R. Acutis M. Bellocchi G. Donatelli M. (2009a). Multi-metric evaluation of the models WARM, CropSyst, and WOFOST for rice. Ecol. Model. 220, 1395–1410. doi: 10.1016/j.ecolmodel.2009.02.017

[B17] Confalonieri R. Rosenmund A. S. Baruth B. (2009b). An improved model to simulate rice yield. Agron. Sustain. Dev. 29, 463–474. doi: 10.1051/agro/2009005

[B18] Dai X. Wang Z. Liu S. Yao Y. Zhao R. Xiang T.. (2022). Hyperspectral imagery reveals large spatial variations of heavy metal content in agricultural soil - A case study of remote-sensing inversion based on Orbita Hyperspectral Satellites (OHS) imagery. J. Clean. Prod. 380, 134878. doi: 10.1016/j.jclepro.2022.134878

[B19] Datt B. (1999). Remote sensing of water content in eucalyptus leaves. Aust. J. Bot. 47, 909–923. doi: 10.1071/BT98042

[B20] Daughtry C. S. T. Walthall C. L. Kim M. S. de Colstoun E. B. McMurtrey J. E. (2000). Estimating corn leaf chlorophyll concentration from leaf and canopy reflectance. Remote Sens. Environ. 74, 229–239. doi: 10.1016/S0034-4257(00)00113-9

[B21] Duan B. Liu Y. Gong Y. Peng Y. Wu X. Zhu R.. (2019). Remote estimation of rice LAI based on Fourier spectrum texture from UAV image. Plant Methods 15, 124. doi: 10.1186/s13007-019-0507-8 31695729 PMC6824110

[B22] Feng X. Tang L. Xu M. (2021). Estimating the biomass of rice by combining GF-1 and RADARSAT-2 data. Arab. J. Geosci. 14, 2124. doi: 10.1007/s12517-021-08545-7

[B23] Gao L. Chai G. Zhang X. (2022). Above-ground biomass estimation of plantation with different tree species using airborne liDAR and hyperspectral data. Remote Sens. 14, 2568. doi: 10.3390/rs14112568

[B24] García-Martínez H. Flores-Magdaleno H. Ascencio-Hernández R. Khalil-Gardezi A. Tijerina-Chávez L. Mancilla-Villa O. R.. (2020). Corn grain yield estimation from vegetation indices, canopy cover, plant density, and a neural network using multispectral and RGB images acquired with unmanned aerial vehicles. Agriculture 10, 277. doi: 10.3390/agriculture10070277

[B25] Gitelson A. A. (2013). Remote estimation of crop fractional vegetation cover: the use of noise equivalent as an indicator of performance of vegetation indices. Int. J. Remote Sens. 34, 6054–6066. doi: 10.1080/01431161.2013.793868

[B26] Gitelson A. A. Andrés V. Arkebauer T. J. Rundquist D. C. Keydan G. (2003). Remote estimation of leaf area index and green leaf biomass in maize canopies. Geophys. Res. Lett. 30, 1248. doi: 10.1029/2002GL016450

[B27] Gitelson A. A. Kaufman Y. J. Merzlyak M. N. (1996). Use of a green channel in remote sensing of global vegetation from EOS-MODIS. Remote Sens. Environ. 58, 289–298. doi: 10.1016/S0034-4257(96)00072-7

[B28] Gitelson A. A. Kaufman Y. J. Stark R. Rundquist D. (2002). Novel algorithms for remote estimation of vegetation fraction. Remote Sens. Environ. 80, 76–87. doi: 10.1016/S0034-4257(01)00289-9

[B29] Gnyp M. L. Miao Y. Yuan F. Ustin S. L. Yu K. Yao Y.. (2014). Hyperspectral canopy sensing of paddy rice aboveground biomass at different growth stages. Field Crops Res. 155, 42–55. doi: 10.1016/j.fcr.2013.09.023

[B30] Gnyp M. L. Yao Y. Yu K. Huang S. Aasen H. Lenz-Wiedemann V. I. S.. (2012). HYPERSPECTRAL ANALYSIS OF RICE PHENOLOGICAL STAGES IN NORTHEAST CHINA. ISPRS Ann. Photogramm. Remote Sens. Spat. Inf. Sci. I–7, 77–82. doi: 10.5194/isprsannals-I-7-77-2012

[B31] Gnyp M. L. Yu K. Aasen H. Yao Y. Huang S. Miao Y.. (2013). Analysis of crop reflectance for estimating biomass in rice canopies at different phenological stages. Photogramm. Fernerkund. Geoinformation 2013, 351–365. doi: 10.1127/1432-8364/2013/0182

[B32] Gobron N. Pinty B. Verstraete M. Widlowski J.-L. (2000). Advanced vegetation indices optimized for up-coming sensors: Design, performance, and applications. IEEE Trans. Geosci. Remote Sens. 38, 2489–2505. doi: 10.1109/36.885197

[B33] Goodwin A. W. Lindsey L. E. Harrison S. K. Paul P. A. (2018). Estimating wheat yield with normalized difference vegetation index and fractional green canopy cover. Crop Forage Turfgrass Manage. 4, 180026. doi: 10.2134/cftm2018.04.0026

[B34] Guevara-Escobar A. Tellez J. Gonzalez-Sosa E. (2005). Use of digital photography for analysis of canopy closure. Agrofor. Syst. 65, 175–185. doi: 10.1007/s10457-005-0504-y

[B35] Haboudane D. Miller J. R. Pattey E. Zarco-Tejada P. J. Strachan I. B. (2004). Hyperspectral vegetation indices and novel algorithms for predicting green LAI of crop canopies: Modeling and validation in the context of precision agriculture. Remote Sens. Environ. 90, 337–352. doi: 10.1016/j.rse.2003.12.013

[B36] Han X. Wei Z. Chen H. Zhang B. Li Y. Du T. (2021). Inversion of winter wheat growth parameters and yield under different water treatments based on UAV multispectral remote sensing. Front. Plant Sci. 12. doi: 10.3389/fpls.2021.609876 PMC8173193 34093601

[B37] Hoffer R. M. (1978). Biological and physical considerations in applying computer-aided analysis techniques to remote sensor data. In Swain P.H. Davis S.M. (Eds.), Remote Sensing: The Quantitative Approach, McGrawHill Book Company, New York , 227–289.

[B38] Huang M. Ibrahim M. Xia B. Zou Y. (2011). Significance, progress and prospects for research in simplified cultivation technologies for rice in China. J. Agric. Sci. 149, 487–496. doi: 10.1017/S0021859610001097 22505773 PMC3320808

[B39] Huete A. Didan K. Miura T. Rodriguez E. P. Gao X. Ferreira L. G. (2002). Overview of the radiometric and biophysical performance of the MODIS vegetation indices. Remote Sens. Environ. 83, 195–213. doi: 10.1016/S0034-4257(02)00096-2

[B40] Huete A. R. (1988). A soil-adjusted vegetation index (SAVI). Remote Sens. Environ. 25, 295–309. doi: 10.1016/0034-4257(88)90106-X

[B41] Hyndman R. J. Koehler A. B. (2006). Another look at measures of forecast accuracy. Int. J. Forecast. 22, 679–688. doi: 10.1016/j.ijforecast.2006.03.001

[B42] Inoue Y. Sakaiya E. Wang C. (2014). Capability of C-band backscattering coefficients from high-resolution satellite SAR sensors to assess biophysical variables in paddy rice. Remote Sens. Environ. 140, 257–266. doi: 10.1016/j.rse.2013.09.001

[B43] Izquierdo-Verdiguier E. Zurita-Milla R. (2020). An evaluation of Guided Regularized Random Forest for classification and regression tasks in remote sensing. Int. J. Appl. Earth Obs. Geoinformation 88, 102051. doi: 10.1016/j.jag.2020.102051

[B44] Ji Y. Chen Z. Cheng Q. Liu R. Li M. Yan X.. (2022). Estimation of plant height and yield based on UAV imagery in faba bean (Vicia faba L.). Plant Methods 18, 26. doi: 10.1186/s13007-022-00861-7 35246179 PMC8897926

[B45] Jiang X. Fang S. Huang X. Liu Y. Guo L. (2021). Rice mapping and growth monitoring based on time series GF-6 images and red-edge bands. Remote Sens. 13, 579. doi: 10.3390/rs13040579

[B46] Jiang Z. Huete A. R. Chen J. Chen Y. Li J. Yan G.. (2006). Analysis of NDVI and scaled difference vegetation index retrievals of vegetation fraction. Remote Sens. Environ. 101, 366–378. doi: 10.1016/j.rse.2006.01.003

[B47] Jiang Z. Huete A. R. Didan K. Miura T. (2008). Development of a two-band enhanced vegetation index without a blue band. Remote Sens. Environ. 112, 3833–3845. doi: 10.1016/j.rse.2008.06.006

[B48] Jordan C. F. (1969). Derivation of leaf-area index from quality of light on the forest floor. Ecology 50, 663–666. doi: 10.2307/1936256

[B49] Karch J. (2020). Improving on adjusted R-squared. Collabra Psychol. 6, 45. doi: 10.1525/collabra.343

[B50] Kawamura K. Asai H. Yasuda T. Khanthavong P. Soisouvanh P. Phongchanmixay S. (2020). Field phenotyping of plant height in an upland rice field in Laos using low-cost small unmanned aerial vehicles (UAVs). Plant Prod. Sci. 23, 452–465. doi: 10.1080/1343943X.2020.1766362

[B51] Kayanan M. Wijekoon P. (2019). Performance of LASSO and Elastic net estimators in Misspecified Linear Regression Model. Ceylon J. Sci. 48, 293. doi: 10.4038/cjs.v48i3.7654

[B52] Kayanan M. Wijekoon P. (2020). Variable selection via biased estimators in the linear regression model. Open J. Stat. 10, 113–126. doi: 10.4236/ojs.2020.101009

[B53] Khan A. M. Osińska M. (2023). Comparing forecasting accuracy of selected grey and time series models based on energy consumption in Brazil and India. Expert Syst. Appl. 212, 118840. doi: 10.1016/j.eswa.2022.118840

[B54] Kolasa-Wiecek A. (2015). Stepwise multiple regression method of greenhouse gas emission modeling in the energy sector in Poland. J. Environ. Sci. 30, 47–54. doi: 10.1016/j.jes.2014.09.037 25872708

[B55] Ku N.-W. Popescu S. C. (2019). A comparison of multiple methods for mapping local-scale mesquite tree aboveground biomass with remotely sensed data. Biomass Bioenergy 122, 270–279. doi: 10.1016/j.biombioe.2019.01.045

[B56] Lee K.-J. Lee B.-W. (2011). Estimating canopy cover from color digital camera image of rice field. J. Crop Sci. Biotechnol. 14, 151–155. doi: 10.1007/s12892-011-0029-z

[B57] Lee Rodgers J. Nicewander W. A. (1988). Thirteen ways to look at the correlation coefficient. Am. Stat. 42, 59–66. doi: 10.2307/2685263

[B58] Li Z. Xin X. Tang H. Yang F. Chen B. Zhang B. (2017). Estimating grassland LAI using the Random Forests approach and Landsat imagery in the meadow steppe of Hulunber, China. J. Integr. Agric. 16, 286–297. doi: 10.1016/S2095-3119(15)61303-X

[B59] Li Z. Zhao Y. Taylor J. Gaulton R. Jin X. Song X.. (2022). Comparison and transferability of thermal, temporal and phenological-based in-season predictions of above-ground biomass in wheat crops from proximal crop reflectance data. Remote Sens. Environ. 273, 112967. doi: 10.1016/j.rse.2022.112967

[B60] Liu Y. Feng H. Sun Q. Yang F. Yang G. (2021). Estimation study of above ground biomass in potato based on UAV digital images with different resolutions. Spectrosc. Spectr. Anal. 41, 1470. doi: 10.3964/j.issn.1000-0593(2021)05-1470-07

[B61] Liu Y. Feng H. Yue J. Fan Y. Bian M. Ma Y.. (2023c). Estimating potato above-ground biomass by using integrated unmanned aerial system-based optical, structural, and textural canopy measurements. Comput. Electron. Agric. 213, 108229. doi: 10.1016/j.compag.2023.108229

[B62] Liu Y. Feng H. Yue J. Fan Y. Jin X. Zhao Y.. (2022a). Estimation of potato above-ground biomass using UAV-based hyperspectral images and machine-learning regression. Remote Sens. 14, 5449. doi: 10.3390/rs14215449

[B63] Liu Y. Feng H. Yue J. Jin X. Fan Y. Chen R.. (2023b). Improved potato AGB estimates based on UAV RGB and hyperspectral images. Comput. Electron. Agric. 214, 108260. doi: 10.1016/j.compag.2023.108260

[B64] Liu Y. Feng H. Yue J. Jin X. Li Z. Yang G. (2022b). Estimation of potato above-ground biomass based on unmanned aerial vehicle red-green-blue images with different texture features and crop height. Front. Plant Sci. 13. doi: 10.3389/fpls.2022.938216 PMC9452666 36092445

[B65] Liu Y. Feng H. Yue J. Li Z. Yang G. Song X.. (2022c). Remote-sensing estimation of potato above-ground biomass based on spectral and spatial features extracted from high-definition digital camera images. Comput. Electron. Agric. 198, 107089. doi: 10.1016/j.compag.2022.107089

[B66] Liu J. Zhu Y. Song L. Su X. Li J. Zheng J.. (2023a). Optimizing window size and directional parameters of GLCM texture features for estimating rice AGB based on UAVs multispectral imagery. Front. Plant Sci. 14. doi: 10.3389/fpls.2023.1284235 PMC10773816 38192693

[B67] Lundberg S. M. Lee S.-I. (2017)A unified approach to interpreting model predictions. In: Advances in neural information processing systems (Long Beach, CA, USA: Curran Associates, Inc). Available online at: https://proceedings.neurips.cc/paper/2017/hash/8a20a8621978632d76c43dfd28b67767-Abstract.html (Accessed August 18, 2023).

[B68] Luo S. Jiang X. Yang K. Li Y. Fang S. (2022). Multispectral remote sensing for accurate acquisition of rice phenotypes: Impacts of radiometric calibration and unmanned aerial vehicle flying altitudes. Front. Plant Sci. 13. doi: 10.3389/fpls.2022.958106 PMC9401905 36035659

[B69] Maimaitijiang M. Sagan V. Sidike P. Maimaitiyiming M. Hartling S. Peterson K. T.. (2019). Vegetation Index Weighted Canopy Volume Model (CVMVI) for soybean biomass estimation from Unmanned Aerial System-based RGB imagery. ISPRS J. Photogramm. Remote Sens. 151, 27–41. doi: 10.1016/j.isprsjprs.2019.03.003

[B70] Mashimbye Z. E. Cho M. A. Nell J. P. De clercq W. P. Van niekerk A. Turner D. P. (2012). Model-based integrated methods for quantitative estimation of soil salinity from hyperspectral remote sensing data: A case study of selected South African soils. Pedosphere 22, 640–649. doi: 10.1016/S1002-0160(12)60049-6

[B71] Milligan G. W. Cooper M. C. (1988). A study of standardization of variables in cluster analysis. J. Classif. 5, 181–204. doi: 10.1007/BF01897163

[B72] Modica G. Messina G. De Luca G. Fiozzo V. Praticò S. (2020). Monitoring the vegetation vigor in heterogeneous citrus and olive orchards. A multiscale object-based approach to extract trees’ crowns from UAV multispectral imagery. Comput. Electron. Agric. 175, 105500. doi: 10.1016/j.compag.2020.105500

[B73] Mostofialmamaleki M. Zulhaidi H. Shafri B. M. Mansor S. Azarkerdar A. (2019). A review: monitoring of rice production by using applications of remote sensing. IOP Conf. Ser. Earth Environ. Sci. 357, 12037. doi: 10.1088/1755-1315/357/1/012037

[B74] Nakajima K. Tanaka Y. Katsura K. Yamaguchi T. Watanabe T. Shiraiwa T. (2023). Biomass estimation of World rice (Oryza sativa L.) core collection based on the convolutional neural network and digital images of canopy. Plant Prod. Sci. 26, 187–196. doi: 10.1080/1343943X.2023.2210767

[B75] Nielsen D. C. Miceli-Garcia J. J. Lyon D. J. (2012). Canopy cover and leaf area index relationships for wheat, triticale, and corn. Agron. J. 104, 1569–1573. doi: 10.2134/agronj2012.0107n

[B76] Otsu N. (1979). A threshold selection method from gray-level histograms. IEEE Trans. Syst. Man Cybern. 9, 62–66. doi: 10.1109/TSMC.1979.4310076

[B77] Panday U. S. Shrestha N. Maharjan S. Pratihast A. K. Shahnawaz Shrestha K. L.. (2020). Correlating the plant height of wheat with above-ground biomass and crop yield using drone imagery and crop surface model, A case study from Nepal. Drones 4, 28. doi: 10.3390/drones4030028

[B78] Peñuelas J. Isla R. Filella I. Araus J. L. (1997). Visible and near-infrared reflectance assessment of salinity effects on barley. Crop Sci. 37, 198–202. doi: 10.2135/cropsci1997.0011183X003700010033x

[B79] Qiu Z. Ma F. Li Z. Xu X. Ge H. Du C. (2021). Estimation of nitrogen nutrition index in rice from UAV RGB images coupled with machine learning algorithms. Comput. Electron. Agric. 189, 106421. doi: 10.1016/j.compag.2021.106421

[B80] Rondeaux G. Steven M. Baret F. (1996). Optimization of soil-adjusted vegetation indices. Remote Sens. Environ. 55, 95–107. doi: 10.1016/0034-4257(95)00186-7

[B81] Roujean J.-L. Breon F.-M. (1995). Estimating PAR absorbed by vegetation from bidirectional reflectance measurements. Remote Sens. Environ. 51, 375–384. doi: 10.1016/0034-4257(94)00114-3

[B82] Rouse J. W. Haas R. H. Schell J. A. Deering D. W. (1973). Monitoring the vernal advancement and retrogradation (green wave effect) of natural vegetation.

[B83] Shen L. Gao M. Yan J. Li Z.-L. Leng P. Yang Q.. (2020). Hyperspectral estimation of soil organic matter content using different spectral preprocessing techniques and PLSR method. Remote Sens. 12, 1206. doi: 10.3390/rs12071206

[B84] Shu M. Li Q. Ghafoor A. Zhu J. Li B. Ma Y. (2023). Using the plant height and canopy coverage to estimation maize aboveground biomass with UAV digital images. Eur. J. Agron. 151, 126957. doi: 10.1016/j.eja.2023.126957

[B85] Sim S. F. Chai M. X. L. Jeffrey Kimura A. L. (2018). Prediction of lard in palm olein oil using simple linear regression (SLR), multiple linear regression (MLR), and partial least squares regression (PLSR) based on fourier-transform infrared (FTIR). J. Chem. 2018, e7182801. doi: 10.1155/2018/7182801

[B86] Simmons S. R. Oelke E. A. Anderson P. M. (1985) Growth and Development Guide for Spring Wheat (St. Paul, MN: University of Minnesota Agricultural Extension Service). Available online at: http://conservancy.umn.edu/handle/11299/165834 (Accessed February 3, 2024).

[B87] Smola A. J. Schölkopf B. (2004). A tutorial on support vector regression. Stat. Comput. 14, 199–222. doi: 10.1023/B:STCO.0000035301.49549.88

[B88] Sun Y. Luo Y. Zhang Q. Xu L. Wang L. Zhang P. (2022). Estimation of crop height distribution for mature rice based on a moving surface and 3D point cloud elevation. Agronomy 12, 836. doi: 10.3390/agronomy12040836

[B89] Varela S. Pederson T. Bernacchi C. J. Leakey A. D. B. (2021). Understanding growth dynamics and yield prediction of sorghum using high temporal resolution UAV imagery time series and machine learning. Remote Sens. 13, 1763. doi: 10.3390/rs13091763

[B90] Vincini M. Frazzi E. D’Alessio P. (2008). A broad-band leaf chlorophyll vegetation index at the canopy scale. Precis. Agric. 9, 303–319. doi: 10.1007/s11119-008-9075-z

[B91] Vu H. L. Ng K. T. W. Richter A. An C. (2022). Analysis of input set characteristics and variances on k-fold cross validation for a Recurrent Neural Network model on waste disposal rate estimation. J. Environ. Manage. 311, 114869. doi: 10.1016/j.jenvman.2022.114869 35287077

[B92] Walter A. Liebisch F. Hund A. (2015). Plant phenotyping: from bean weighing to image analysis. Plant Methods 11, 14. doi: 10.1186/s13007-015-0056-8 25767559 PMC4357161

[B93] Wan L. Cen H. Zhu J. Zhang J. Zhu Y. Sun D.. (2020). Grain yield prediction of rice using multi-temporal UAV-based RGB and multispectral images and model transfer – a case study of small farmlands in the South of China. Agric. For. Meteorol. 291, 108096. doi: 10.1016/j.agrformet.2020.108096

[B94] Wang J. Huang J. Gao P. Wei C. Mansaray L. R. (2016). Dynamic mapping of rice growth parameters using HJ-1 CCD time series data. Remote Sens. 8, 931. doi: 10.3390/rs8110931

[B95] Wang D. Li R. Zhu B. Liu T. Sun C. Guo W. (2022a). Estimation of wheat plant height and biomass by combining UAV imagery and elevation data. Agriculture 13, 9. doi: 10.3390/agriculture13010009

[B96] Wang Q. Lu X. Zhang H. Yang B. Gong R. Zhang J.. (2023a). Comparison of machine learning methods for estimating leaf area index and aboveground biomass of cinnamomum camphora based on UAV multispectral remote sensing data. Forests 14, 1688. doi: 10.3390/f14081688

[B97] Wang Z. Ma Y. Chen P. Yang Y. Fu H. Yang F.. (2022b). Estimation of rice aboveground biomass by combining canopy spectral reflectance and unmanned aerial vehicle-based red green blue imagery data. Front. Plant Sci. 13. doi: 10.3389/fpls.2022.903643 PMC9197132 35712565

[B98] Wang X. Xu G. Feng Y. Peng J. Gao Y. Li J.. (2023b). Estimation model of rice aboveground dry biomass based on the machine learning and hyperspectral characteristic parameters of the canopy. Agronomy 13, 1940. doi: 10.3390/agronomy13071940

[B99] Wang P. Zhang Z. Song X. Chen Y. Wei X. Shi P.. (2014). Temperature variations and rice yields in China: historical contributions and future trends. Clim. Change 124, 777–789. doi: 10.1007/s10584-014-1136-x

[B100] Willkomm M. Bolten A. Bareth G. (2016). NON-DESTRUCTIVE MONITORING OF RICE BY HYPERSPECTRAL IN-FIELD SPECTROMETRY AND UAV-BASED REMOTE SENSING: CASE STUDY OF FIELD-GROWN RICE IN NORTH RHINE-WESTPHALIA, GERMANY. Int. Arch. Photogramm. Remote Sens. Spat. Inf. Sci. XLI-B1, 1071–1077. doi: 10.5194/isprs-archives-XLI-B1-1071-2016

[B101] Xin Z. Jun S. Yan T. Quansheng C. Xiaohong W. Yingying H. (2020). A deep learning based regression method on hyperspectral data for rapid prediction of cadmium residue in lettuce leaves. Chemom. Intell. Lab. Syst. 200, 103996. doi: 10.1016/j.chemolab.2020.103996

[B102] Xu T. Wang F. Shi Z. Xie L. Yao X. (2023). Dynamic estimation of rice aboveground biomass based on spectral and spatial information extracted from hyperspectral remote sensing images at different combinations of growth stages. ISPRS J. Photogramm. Remote Sens. 202, 169–183. doi: 10.1016/j.isprsjprs.2023.05.021

[B103] Xu T. Wang F. Xie L. Yao X. Zheng J. Li J.. (2022b). Integrating the textural and spectral information of UAV hyperspectral images for the improved estimation of rice aboveground biomass. Remote Sens. 14, 2534. doi: 10.3390/rs14112534

[B104] Xu L. Zhou L. Meng R. Zhao F. Lv Z. Xu B.. (2022a). An improved approach to estimate ratoon rice aboveground biomass by integrating UAV-based spectral, textural and structural features. Precis. Agric. 23, 1276–1301. doi: 10.1007/s11119-022-09884-5

[B105] Xue J. Su B. (2017). Significant remote sensing vegetation indices: A review of developments and applications. J. Sens. 2017, 1–17. doi: 10.1155/2017/1353691

[B106] Yang S. Feng Q. Liang T. Liu B. Zhang W. Xie H. (2018). Modeling grassland above-ground biomass based on artificial neural network and remote sensing in the Three-River Headwaters Region. Remote Sens. Environ. 204, 448–455. doi: 10.1016/j.rse.2017.10.011

[B107] Yang K. Mo J. Luo S. Peng Y. Fang S. Wu X.. (2023). Estimation of rice aboveground biomass by UAV imagery with photosynthetic accumulation models. Plant Phenomics 5, 56. doi: 10.34133/plantphenomics.0056 PMC10238111 37273463

[B108] Yao R. Yang J. Gao P. Zhang J. Jin W. (2013). Determining minimum data set for soil quality assessment of typical salt-affected farmland in the coastal reclamation area. Soil Tillage Res. 128, 137–148. doi: 10.1016/j.still.2012.11.007

[B109] Yu Q. Liu J. Zhang Y. Li J. (2002). Simulation of rice biomass accumulation by an extended logistic model including influence of meteorological factors. Int. J. Biometeorol. 46, 185–191. doi: 10.1007/s00484-002-0141-3 12242474

[B110] Yu D. Zha Y. Sun Z. Li J. Jin X. Zhu W.. (2023). Deep convolutional neural networks for estimating maize above-ground biomass using multi-source UAV images: a comparison with traditional machine learning algorithms. Precis. Agric. 24, 92–113. doi: 10.1007/s11119-022-09932-0

[B111] Yue J. Yang G. Tian Q. Feng H. Xu K. Zhou C. (2019). Estimate of winter-wheat above-ground biomass based on UAV ultrahigh-ground-resolution image textures and vegetation indices. ISPRS J. Photogramm. Remote Sens. 150, 226–244. doi: 10.1016/j.isprsjprs.2019.02.022

[B112] Zadoks J. C. Chang T. T. Konzak C. F. (1974). A decimal code for the growth stages of cereals. Weed Res. 14, 415–421. doi: 10.1111/j.1365-3180.1974.tb01084.x

[B113] Zhai W. Li C. Fei S. Liu Y. Ding F. Cheng Q.. (2023). CatBoost algorithm for estimating maize above-ground biomass using unmanned aerial vehicle-based multi-source sensor data and SPAD values. Comput. Electron. Agric. 214, 108306. doi: 10.1016/j.compag.2023.108306

[B114] Zhang S.-H. He L. Duan J.-Z. Zang S.-L. Yang T.-C. Schulthess U. R. S.. (2024). Aboveground wheat biomass estimation from a low-altitude UAV platform based on multimodal remote sensing data fusion with the introduction of terrain factors. Precis. Agric. 25, 119–145. doi: 10.1007/s11119-023-10062-4

[B115] Zhang X. Liu C.-A. (2023). Model averaging prediction by K-fold cross-validation. J. Econom. 235, 280–301. doi: 10.1016/j.jeconom.2022.04.007

[B116] Zhang J. Tian H. Wang D. Li H. Mouazen A. M. (2020). A novel approach for estimation of above-ground biomass of sugar beet based on wavelength selection and optimized support vector machine. Remote Sens. 12, 620. doi: 10.3390/rs12040620

[B117] Zhang Q. Zhang W. Li T. Sun W. Yu Y. Wang G. (2017). Projective analysis of staple food crop productivity in adaptation to future climate change in China. Int. J. Biometeorol. 61, 1445–1460. doi: 10.1007/s00484-017-1322-4 28247124

[B118] Zhang X. Zhang K. Sun Y. Zhao Y. Zhuang H. Ban W.. (2022). Combining spectral and texture features of UAS-based multispectral images for maize leaf area index estimation. Remote Sens. 14, 331. doi: 10.3390/rs14020331

[B119] Zheng L. Chen Q. Tao J. Zhang Y. Lei Y. Zhao J.. (2023). Estimation of aboveground biomass for winter wheat at the later growth stage by combining digital texture and spectral analysis. Agronomy 13, 865. doi: 10.3390/agronomy13030865

[B120] Zhou S. Xu L. Chen N. (2023). Rice yield prediction in hubei province based on deep learning and the effect of spatial heterogeneity. Remote Sens. 15, 1361. doi: 10.3390/rs15051361

[B121] Zhu W. Rezaei E. E. Nouri H. Sun Z. Li J. Yu D.. (2023). UAV flight height impacts on wheat biomass estimation via machine and deep learning. IEEE J. Sel. Top. Appl. Earth Obs. Remote Sens. 16, 7471–7485. doi: 10.1109/JSTARS.2023.3302571

[B122] Zou H. Hastie T. (2005). Regularization and variable selection via the elastic net. J. R. Stat. Soc Ser. B Stat. Methodol. 67, 301–320. doi: 10.1111/j.1467-9868.2005.00503.x

